# Occurrence, Fate, and Related Health Risks of PFAS in Raw and Produced Drinking Water

**DOI:** 10.1021/acs.est.2c06015

**Published:** 2023-02-13

**Authors:** Mohammad Sadia, Ingeborg Nollen, Rick Helmus, Thomas L. ter Laak, Frederic Béen, Antonia Praetorius, Annemarie P. van Wezel

**Affiliations:** † Institute for Biodiversity and Ecosystem Dynamics, University of Amsterdam, P.O. Box 94240, 1090 GE Amsterdam, The Netherlands; ‡ KWR Water Research Institute, P.O. Box 1072, 3430 BB Nieuwegein, The Netherlands

**Keywords:** PFAS, exposure assessment, water quality, PFAS isomers, drinking water

## Abstract

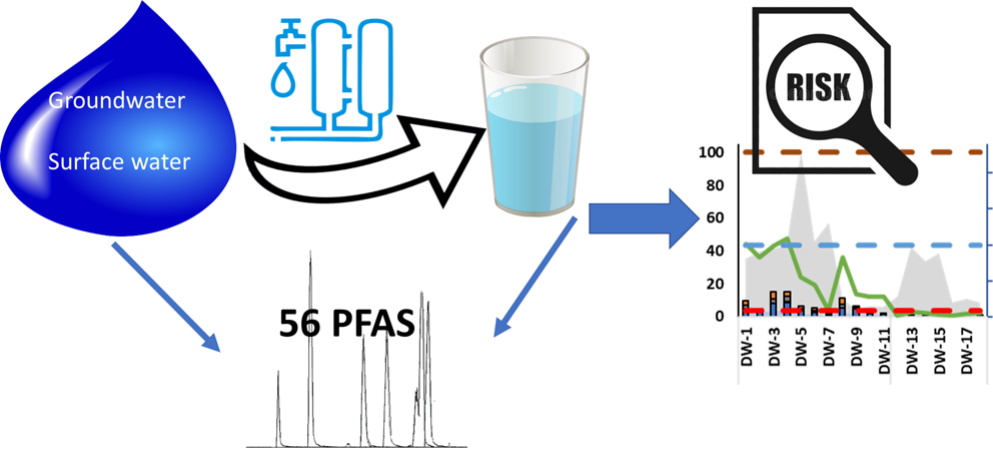

This study investigates human exposure to per- and polyfluoroalkyl substances (PFAS) via drinking water and evaluates human health risks. An analytical method for 56 target PFAS, including ultrashort-chain (C2–C3) and branched isomers, was developed. The limit of detection (LOD) ranged from 0.009 to 0.1 ng/L, except for trifluoroacetic-acid and perfluoropropanoic-acid with higher LODs of 35 and 0.24 ng/L, respectively. The method was applied to raw and produced drinking water from 18 Dutch locations, including groundwater or surface water as source, and applied various treatment processes. Ultrashort-chain (300 to 1100 ng/L) followed by the group of perfluoroalkyl-carboxylic-acids (PFCA, ≥C4) (0.4 to 95.1 ng/L) were dominant. PFCA and perfluoroalkyl-sulfonic-acid (≥C4), including precursors, showed significantly higher levels in drinking water produced from surface water. However, no significant difference was found for ultrashort PFAS, indicating the need for groundwater protection. Negative removal of PFAS occasionally observed for advanced treatment indicates desorption and/or degradation of precursors. The proportion of branched isomers was higher in raw and produced drinking water as compared to industrial production. Drinking water produced from surface water, except for a few locations, exceed non-binding provisional guideline values proposed; however, all produced drinking waters met the recent soon-to-be binding drinking-water-directive requirements.

## Abstract 2

An analytical method was optimized to analyze 56-PFAS in raw and produced drinking water in the Netherlands to evaluate human health risks.

## 1 Introduction

Sustainable access to safe drinking water is recognized as a fundamental human right and formulated as one of the Sustainable Development Goals (SDGs).^1^ Therefore, water pollution and the release of hazardous chemicals are to be minimized, as the presence of anthropogenic chemicals in drinking water may pose risks to human health. In developed countries, occurrence levels of chemicals in drinking water generally are well below levels of concern for human health.^2^

Per- and polyfluoroalkyl substances (PFAS) are a wide range of modern chemical substances raising increasing concern for human and environmental health.^3−5^ They are defined as fluorinated substances that contain at least a perfluorinated methyl group (−CF3) or a perfluorinated methylene group (−CF2−),^6^ a definition that covers a broad range of chemicals.^7^ The occurrence of PFAS in drinking waters is a consequence of their widespread use and environmental fate.^8,9^ High production and use volumes combined with very high persistence, mobility, and bio-accumulative properties lead to strong concerns about PFAS already exceeding planetary boundaries.^10^ As a result, increasing levels of PFAS have been detected in the environment, food and consumer products, human milk and blood, and drinking and surface water.^11−18^ Before 2002, PFAS were produced via electrochemical fluorination (ECF) and more recently via telomerization reactions.^19^ Telomerization results in substances only consisting of linear alkyl chains, whereas ECF results in a mixture of branched and linear isomers.^20^ The physical–chemical properties of the linear and branched isomer forms differ, translating into differences in their environmental fate such as sorption, transformation, and bioaccumulation.^21^ While the occurrence and fate of long-chain PFAS, especially the linear form of perfluoro-octane sulfonic acid (PFOS) and perfluoro-octanoic acid (PFOA), are relatively well studied, much less information is available on ultrashort PFAS (C2–C3) and branched isomers. Due to restrictions and regulations of long-chain PFAS and possibly a future broad PFAS restriction, a transition to non-PFAS alternatives is currently underway.^22^ However, in the absence of a thorough evaluation of the new alternatives, regrettable substitutions may occur.^23^ At current, alternatives may also still include other emerging PFAS, for example, short (C4–C6) to ultrashort chain PFAS, or PFAS precursors which break down to short and ultrashort PFAS.^24^ These PFAS alternatives are also recognized as very persistent and very mobile in the environment and likely to lead to future groundwater contamination.^25^

Toxicological studies are available only for a relatively small number of PFAS,^26^ and often it is not specified whether linear, branched, or a mixture of isomer forms of PFAS were tested.^27^ The European Food Safety Authority (EFSA) stated that drinking water and food are the main sources of human exposure to PFAS and recommended a total weekly intake (TWI) of 4.4 ng/kg body weight for the sum of 4 PFAS [so called “4-EFSA”, namely, PFOA, perfluoro-nonanoic acid (PFNA), perfluoro-hexane sulfonic acid (PFHxS), and PFOS].^27^ This TWI is significantly more stringent compared to earlier assessments,^28,29^ and effects on the immune system were considered the most critical end-point for risk assessment. For safe drinking water concentrations following standard assumptions—that is, an allocation factor of 20%, intake of 2 L/d, and 60 kg body weight^30^—this TWI would translate to 3.7 ng/L for the sum of these four EFSA-PFAS. In parallel with the publication of the EFSA opinion, the EU drinking water directive (DWD) was revised and adopted and EU Member States have until 2023 to transpose it into national legislation,^31^ including drinking water quality standards for a defined sum of 20 PFAS at 100 ng/L or for total PFAS at 500 ng/L. A recent study by Bil et al. proposes to use relative potency factors (RPFs), which normalizes the dose of each PFAS, according to its potency, to PFOA as an index compound. RPFs facilitate a weighted risk assessment for PFAS mixtures, and have been recently discussed with regard to their robustness.^32,33^

In the Netherlands, drinking water is produced from surface water (∼40%), including riverbank and dune filtration, or from groundwater (∼60%) as raw water sources.^34^ All these sources might be contaminated by a variety of PFAS.^35^ Conventional treatment such as flocculation, aeration, and sand filtration, as well as advanced treatment based on size separation [e.g., reverse osmosis (RO)], oxidation, or sorption processes, is used to remove pollutants from drinking water.^34,36^ Despite the treatment methods, different PFAS have been found in the produced drinking water in the Netherlands and other countries.^35,37^

This study aims to investigate the occurrence of PFAS in raw and produced drinking water and determine removal efficiencies during drinking water treatment. This is to assess the human exposure to PFAS via drinking water, including exposure to rarely mentioned very polar ultrashort chain and PFAS branched isomers, and to assess related health risks based on the latest evaluations by non-binding EFSA and binding DWD and by application of RPFs. To understand the vulnerability of different drinking water sources to PFAS contamination and the removal efficiency by various drinking water treatment schemes, raw and produced drinking water from 18 different full-scale production locations in the Netherlands are investigated. A series of 56 PFAS ranging from ultrashort-chain PFAS (C2–C3) and a variety of precursors and various medium to long-chain PFAS (C4–C14) were studied.

## 2 Material and Methods

### 2.1 Standards and Chemicals

Native and isotopic mass labeled standards were purchased from Wellington Laboratories (Guelph, Canada), except for *n*-deuteriomethylperfluoro-1-*n*-octanesulfonamidoacetic acid-*d*_3_ (*N*-MeFOSAA-*d*_3_, >99%) and *n*-ethylperfluoro-1-*n*-octanesulfonamidoacetic acid-*d*_5_ (*N*-EtFOSAA-*d*_5_, >99%) that were purchased from Chiron (Trondheim, Norway), trifluoroacetic acid (TFA, >99%) and perfluoropropanoic acid (PFPrA, >97%) that were purchased from Sigma-Aldrich (Zwijndrecht, Netherlands), perfluoroethane sulfonic acid (PFEtS, >98%) that was purchased from Kanto Chemical (Japan), and *n*-methylperfluorobutanesulfonamide (>97%) that was purchased from Apollo Scientific (Manchester, United Kingdom). The full list of standards is given in Table S1 with details on classification for each PFAS. Milli-Q water was used throughout the experiments. Liquid chromatography (LC)–mass spectrometry grade methanol and acetonitrile were acquired from Biosolve Chimie (Dieuze, France). Ammonium acetate (≥99%) and glacial acetic acid (≥99%) were purchased from Sigma-Aldrich, ammonia solution (25%, analytical reagent grade) was acquired from Fisher Scientific (Massachusetts, United States).

### 2.2 Sample Collection

Raw and produced drinking water samples were collected from 18 different drinking water treatment plant locations in the Netherlands (Table 1). The locations were chosen based on the type of source water (surface water: locations 1–11; and groundwater: locations 12–18), type of treatment scheme (advanced 1–11 and conventional 12–18), and the proximity to known sources for PFAS emissions such as a fluorochemical plant, landfills, or firefighting training sites (details in Table 1). Those locations were not representative for the entire drinking water situation in the Netherlands and were kept anonymously labeled at the request of the drinking water companies. This study represents 11 out of 34 production locations based on surface water sources and 6 out of 187 production locations based on groundwater sources. Grab sampling was performed in triplicate by the drinking water company or an accredited drinking water laboratory in March–April 2021. Raw water samples were collected at the drinking water treatment plant’s entry point, and drinking water samples were collected at the final step of treatment before distribution into the network. To ensure sample integrity and to minimize contamination, 2 L HDPE bottles were precleaned with water and methanol before sampling. The samples were directly stored at 4 °C until analysis, which was performed within 2–4 weeks of sampling.

**Table 1:** Information About the Sampling Location and Drinking Water Production Sites^a^

source type	sample code	treatment process	source typology	possible contamination source
surface water	1	advanced (PAC)	river (Meuse)	airport (firefighting training)
	2	advanced (UV/GAC)	river (Rhine)	
	3	advanced (PAC)	river (Meuse)	
	4	advanced (PAC)	river (Meuse)	
	5	advanced (UV/GAC)	river (Meuse)	
	6	advanced (UV/GAC)	river (Meuse)	fluorochemical production plants
	7	advanced (GAC)	dune filtration	airport (firefighting training)
	8	advanced (GAC)	dune filtration	
	9	advance (ozone/GAC)	lake	
	10	advanced (RO/GAC)^b^	river (Rhine)	fluorochemical production plants
	11	advanced (GAC)	river (Rhine)	fluorochemical production plants
groundwater	12	conventional	confined	
	13	conventional	confined	historical landfill contamination
	14	conventional	confined	airport (firefighting training)
	15	conventional	confined	
	16	conventional	confined	
	17	conventional	confined	airport (firefighting training)
	18	conventional	confined	

a PAC: powder active carbon; GAC: granular active carbon; UV: ultraviolet; RO: reverse osmosis.

b The RO-permeate was mixed with raw water before conventional treatment.

### 2.3 Sample Preparation

Prior to extraction, the samples were sonicated for 10 min in their original HDPE bottle, and then, 1 L was weighed into new precleaned HDPE containers and extracted using a weak anion exchange Oasis (WAX) solid phase extraction (SPE) cartridge according to the standard EPA method 537.^38^ The sample was adjusted to pH 4 using acetic acid and spiked with 10 μL of mass-labeled extraction standard (ES; 0.1–0.2 ng/μL). SPE was performed by loading 1 L sample on WAX-SPE cartridge (3 mL, 60 mg, 30 μm; Waters Corporation Milford, USA). The cartridges were preconditioned by subsequently adding 3 mL of 0.1% ammonium hydroxide in methanol, 3 mL of methanol, and finally 3 mL of Milli-Q water. After loading the sample, the cartridges were washed with 3 mL of ammonium acetate buffer solution at pH 4. The cartridges were then dried for 20 min under vacuum, then elution was performed with 3 mL of 0.1% ammonium hydroxide in methanol. The extract was evaporated under a gentle stream of high-purity nitrogen to 75 μL and then 175 μL 0.05% acetic acid in water and 5 μL of mass-labeled injection standard solution (IS; 0.1 ng/μL) were added. The 250 μL extract was vortex-mixed, centrifuged (5 min at 4000 rpm), and then transferred to an LC vial for further chemical analysis.

### 2.4 Chemical Analysis

To detect trace levels of PFAS in raw and produced drinking water in the pg/L range, method optimization was necessary, aiming at sufficient selectivity to separate co-eluting interferences as well as linear and branched PFAS. Furthermore, the aim was a comprehensive method to cover our wide range of 56 target PFAS (Table S1), ranging from ultrashort-chain PFAS (e.g., TFA) to long-chain PFAS (e.g., PFTeDA) in one single run. Only for analyte HPFO-DA (or Gen-X) a separate procedure was used.

Chromatographic behavior of the target PFAS in a reference standard solution (4 ng/mL) was examined. Different chromatographic separation columns [Kinetex F5 and Biphenyl from Phenomenex (United States), mixed-mode WAX-1 high-performance LC (HPLC) column from Fisher Scientific, CSH C18 column from Waters Corporation Milford], different mobile phase solvents (methanol, acetonitrile) at different pH values between 3 and 11 using mobile phase additives (ammonium acetate, acetic acid, ammonia solution, and 1-methylpiperidine) were investigated, see Figure S1. Furthermore, ionization using different ion sources [electro spray ionization (ESI) and ion booster electro spray ionization (IB-ESI)] were compared.

In our optimized method, the chemical analysis was performed on a Nexera UHPLC system (Shimadzu, Kyoto, Japan) coupled to a Bruker maXis 4 G q-TOF-high-resolution mass spectrometer (HRMS), upgraded with a HD collision cell and equipped with IB-ESI source. Mass spectra were recorded in both positive and negative modes in separate runs with a range of 50–1500 *m*/*z* and a 2 Hz sampling rate. MS setup was as follows: nebulizer gas 4 Bar, drying gas flow 3 L/min, dry gas temp 200 °C, capillary voltage 1000 V, end plate offset 400 V.

Aliquots of 10 μL were injected into an Acquity UPLC CSH C18 column (130 Å, 2.1 × 150 mm, 1.7 μm). The flow rate was set at 0.2 mL/min and the column temperature was set at 50 °C. The mobile phase consisted of 0.05% acetic acid in water (A) and 0.05% acetic acid in acetonitrile (B). The eluent gradient started at 20% and was increased to 100% B using a linear ramp until 23 min, held for 3 min, and then reverted to initial conditions of 20% B. The system was then allowed to re-equilibrate for 5 min before the next sample was injected.

Internal mass calibration was carried out automatically for each analysis to ensure good mass accuracy regardless of the total analysis time. This was achieved by infusing a 50 μM sodium acetate solution in a water/methanol mixture (1:1, v/v), with a loop injection of 20 μL at the beginning of the analysis (0.1–0.5 min). The sodium acetate cluster provided 14 points of calibration ranging from *m*/*z* 59 to *m*/*z* 1207, and at least eight points (standard deviation ≤ 0.5 ppm) were taken for the mass calibration of each sample.

For the analyte HPFO-DA (or Gen-X), the HPLC system (Shimadzu Prominence II XR, Kyoto, Japan) connected to a tandem mass spectrometer (4000 QTrap, Applied Biosystems, Toronto, Canada) operating in the negative ionization mode with scheduled MRM was used. Quantification was based on relative response factor using corresponding extraction and injection mass-labeled standards. Further details about the quantification and quality control can be found in the Supporting Information.

### 2.5 Data Analysis and Method Validation

To check for significant differences in the concentration of various PFAS classes between different sources (surface water or groundwater), and the differences between raw and produced drinking waters, a Mann–Whitney test was performed (*p* < 0,05), with no data below limit of detection (LOD) regarding PFAS classes. Individual PFAS below LOD were replaced with LOD/2 in further statistical analysis. To investigate the difference between branched to the sum of linear and branched contributions, samples with no detection of the linear and branched isomers were excluded to avoid a statistical bias in the results. The drinking water treatment efficiency was evaluated only for surface water, given the low PFAS concentration (pg/L) measured in groundwater-based samples.

For the human health risk assessment, the sum of the linear and branched isomers was considered for all investigated PFAS. The PFOA equivalent (PEQ) was calculated as described by Bil et al.,^32^ and based on the reported RPFs in three different studies^32,33,39^ (list of RPFs provided in Table S2), summing all PEQ for PFAS and compared to the equivalent safe level of the EFSA of 3.7 ng/L.

Method validation: the validation of this method was performed in accordance with the Eurachem guideline on method validation^40^ in terms of LOD, selectivity, recovery, linearity, reproducibility, matrix effect, accuracy, and precision.

The LOD was determined by using the average of the analyte concentration in the procedural blanks plus three times the standard deviation, and in the case of no detection of targeted PFAS in the procedure blank the LOD defined as the lowest point in the calibration curve.^14^ The method selectivity was conducted by comparing the chromatograms of standard in the pure solvent with that of spiked water (quality control sample). Recovery was evaluated based on the measured amount of mass-labeled ES spiked before extraction with the injection standard spiked after extraction multiplied by 100%. Linearity was evaluated by an external calibration curve consisting of a concentration series of 50, 100, 150, 300, 500, 800, 1000, 1200, 2000, 3000, and 5000 pg with a fixed amount of extraction and injection standard added (correlation coefficients *R* > 0.99), at least eight points were taken to obtain linearity *R* > 0.99. The possible matrix effect on the ionization was evaluated by comparison of the peak area for injection standards in the sample and those in a pure solvent. The accuracy and method reproducibility were evaluated by spiked water (2 ng/L) in triplicate analyses using the percentage relative standard deviation (RSD % < 20%). The accuracy was evaluated by comparing the concentration with theoretical levels after subtraction of endogenous present concentrations. Instrument carryover was evaluated by injecting solvent (instrument blank) after each standard injection.

## 3 Results and Discussion

### 3.1 Method Optimization and Validation

The CSH C18 and mixed-mode WAX-1 columns showed a good retainment of all PFAS including the ultrashort chain ones. Both columns did not give satisfactory results for polyfluorinated phosphate monoesters (mono-PAPs) (e.g., PFHxPA, PFOPA). The addition of 1-methyl-piperidine as an ion-pairing agent to the mobile phase^41^ produced high signal suppression and inefficient separation for PFAS in both columns. Using the mixed-mode WAX-1 column requires the use of a high level of ammonium acetate (>20 mM) as a mobile phase additive to ensure elution of all target PFAS,^42^ leading to high suppression in our instrument (Bruker maXis 4G q-TOF-HRMS).

**Figure 1:**
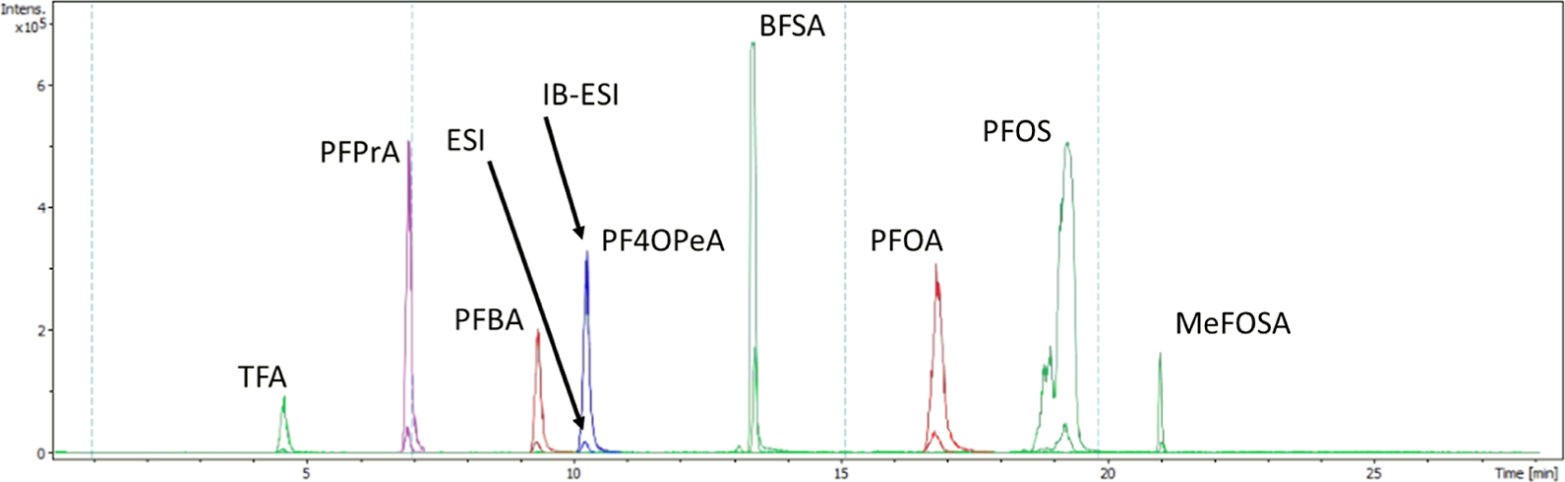
LC–HRMS chromatogram showing the different PFAS after injection of a 4 ng/mL PFAS mixture using the described analytical method. Results for ESI and IB-ESI are shown.

The CSH C18 column with 0.05% acetic acid in acetonitrile showed a sufficient separation for the ultrashort chain, long chain PFAS, and isomer branched PFAS (Figure 1). A limitation of the CSH C18 column is insufficient retention for the mono-PAPs, for which the signal was observed all over the running time.

IB-ESI was ultimately chosen as it not only leads to enhanced PFAS ionization as compared to ESI (Figure 1) but also enhances the matrix ionization and causes matrix effects, so optimized sample extraction was necessary. The matrix effect was strongly influenced by reducing the size of WAX-SPE sorbent. Meanwhile, reducing the amount of sorbed matrix to the sorbent resulted in an improvement of the mass labeled response in the drinking water extract as compared to the pure solvent, from less than 10% with 250 mg of sorbent to 58–127% in the case of 60 mg (for details on individual response see Figure S3). The optimized method yielded a satisfactory result by detecting trace levels of PFAS in drinking water in the pg/L range with a sufficient chromatographical separation of very polar PFAS such as TFA. All procedural blanks showed no detected level of targeted PFAS except for TFA and PFPrA with average contamination levels of 17.33 and 0.17 ng/L, respectively. The method LOD ranged from 0.09 to 0.1 ng/L except for two ultrashort chain PFAS, namely TFA and PFPrA, which had LODs of 35.4 and 0.24 ng/L, respectively (Table S1). This was not surprising due to the challenges in measuring these two compounds as described, for example, by Björnsdotter and Ateia.^25,43^ The total method average recoveries for mass-labeled standards ranged from 47 to 198%, details on individual recoveries are presented in Figure S2. The method reproducibility was acceptable with an RSD < 20% for all investigated PFAS. The method accuracy presented in Table S1, ranged between 70 and 120% for all PFAS standards.

### 3.2 PFAS Concentration in Raw and Produced Drinking Water

**Figure 2:**
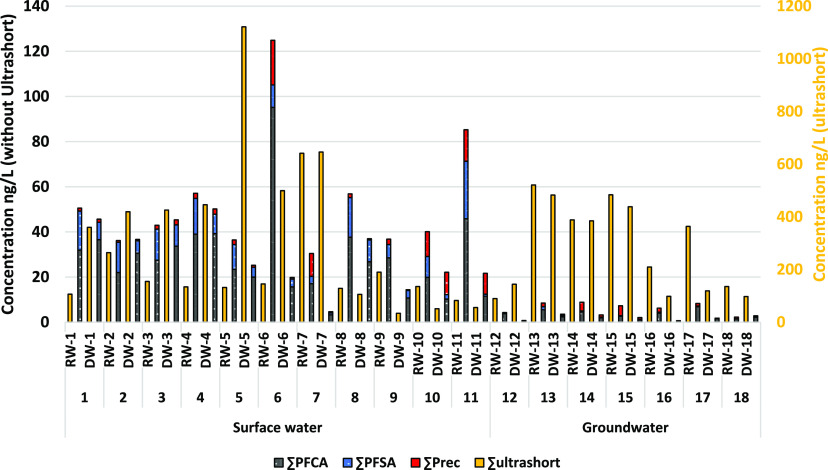
Occurrence of different PFAS classes in raw water (RW) and produced drinking water (DW) for the various sampling locations, concentration (ng/L) on the primary axis as depicted without the dominant ultrashort PFAS, concentration (ng/L) on the secondary axis for the ultrashort PFAS. PFAS class: ultrashort chain PFAS (C2–C3), PFCA: perfluoro-carboxylic acids (C4–C14) PFSA: perfluoro-sulfonic acids (C4–C10), Prec: variety of precursors (C4–C24).

In general raw surface waters contain relatively high levels for the sum of all 56 PFAS monitored in this study (50–1150 ng/L, Figure 2, Tables 2 and S3), reflecting their widespread use, mobility, and persistence. Groundwater shows lower levels of the sum of all 56 PFAS (90–530 ng/L, Figure 2, Tables 2 and S3).

**Table 2:** Descriptive Statistics (Mean, Median, and Detection Frequency Above LOD) for Drinking Water and Raw Water Regarding Individual Targeted PFAS Grouped by Water Sources^a^

	surface water (*n* = 11)						groundwater (*n* = 7)					
	drinking water			raw water			drinking water			raw water		
	mean [SD]	median [min, max]	detection	mean [SD]	median [min, max]	detection	mean [SD]	median [min, max]	detection	mean [SD]	median [min, max]	detection
TFA	351.77 [305.1]	359.86 [33.56, 1104.6]	11	191.69 [149.17]	135.15 [82.44, 641.34]	11	245.31 [167.2]	134.04 [88.44, 482.95]	7	310.17 [157.73]	352.49 [87.77, 520.93]	7
PFPrA	26.59 [25.91]	16.74 [0.12, 65.55]	9	0.39 [0.62]	0.12 [0.12, 2.18]	2	6.8 [9.7]	0.12 [0.12, 28.39]	3	2.78 [3.75]	1.41 [0.12, 11.13]	4
PFPrS	0.11 [0.06]	0.09 [0.03, 0.2]	9	0.07 [0.04]	0.07 [0.03, 0.13]	7	<LOD	<LOD	0	0.03 [0.02]	0.03 [0.03, 0.07]	1
PFEtS	<LOD	<LOD	0	<LOD	<LOD	0	<LOD	<LOD	0	<LOD	<LOD	0
∑ultrashort	378.46 [309.26]	419.14 [33.76, 1121.43]	11	192.16 [149.05]	135.3 [82.59, 641.49]	11	252.12 [161.54]	143.82 [97.43, 483.07]	7	312.98 [157.22]	363.64 [89.25, 521.07]	7
PFBA	7.26 [3.87]	7.9 [1.27, 13.41]	11	6.75 [4.05]	5.12 [2.54, 14.95]	11	0.53 [0.36]	0.4 [0.13, 1.17]	7	1.35 [1.25]	1.04 [0.03, 3.69]	5
PFPeA	3.5 [2.18]	3.75 [0.29, 6.89]	11	2.78 [1.36]	2.82 [0.03, 4.66]	10	0.13 [0.11]	0.1 [0.03, 0.3]	4	0.28 [0.18]	0.31 [0.03, 0.54]	5
PFHxA	4.07 [3.04]	3.34 [0.3, 8.8]	11	3.64 [1.74]	3.66 [1.2, 6.19]	11	0.15 [0.12]	0.15 [0.03, 0.35]	4	0.39 [0.1]	0.4 [0.19, 0.54]	7
PFHpA	2.39 [1.9]	2.01 [0.24, 5.68]	11	2.28 [1.15]	1.83 [0.83, 4.22]	11	0.1 [0.07]	0.1 [0.03, 0.22]	4	0.2 [0.11]	0.23 [0.03, 0.33]	6
L-PFOA	3.37 [2]	3.19 [0.74, 7.17]	11	11.11 [12.67]	5.62 [2.44, 41.97]	11	0.38 [0.26]	0.34 [0.06, 0.87]	7	0.71 [0.43]	0.69 [0.1, 1.27]	7
Br-PFOA	0.7 [0.45]	0.5 [0.09, 1.52]	11	1.3 [1.33]	0.86 [0.3, 4.24]	11	0.06 [0.04]	0.05 [0.03, 0.15]	4	0.07 [0.06]	0.03 [0.03, 0.2]	3
∑PFOA	4.08 [2.42]	3.55 [0.83, 8.7]	11	12.41 [13.95]	6.23 [2.82, 45.83]	11	0.43 [0.31]	0.36 [0.07, 1.03]	7	0.79 [0.47]	0.75 [0.13, 1.47]	7
PFNA	0.22 [0.12]	0.26 [0.03, 0.4]	9	0.36 [0.16]	0.35 [0.09, 0.75]	11	0.05 [0.03]	0.03 [0.03, 0.09]	3	0.04 [0.03]	0.03 [0.03, 0.1]	2
PFDA	0.13 [0.08]	0.14 [0.03, 0.3]	8	0.27 [0.2]	0.24 [0.05, 0.81]	11	0.1 [0.09]	0.08 [0.03, 0.28]	4	0.19 [0.22]	0.07 [0.03, 0.64]	4
PFUdA	0.07 [0]	0.07 [0.07, 0.07]	1	<LOD	<LOD	0	<LOD	<LOD	0	0.03 [0.02]	0.03 [0.03, 0.07]	1
PFDoA	<LOD	<LOD	0	0.05 [0.04]	0.03 [0.03, 0.16]	2	<LOD	<LOD	0	0.04 [0.02]	0.03 [0.03, 0.09]	2
PFTrDA	<LOD	<LOD	0	<LOD	<LOD	0	<LOD	<LOD	0	<LOD	<LOD	0
PFTeDA	<LOD	<LOD	0	0.61 [1.02]	0.03 [0.03, 3.15]	3	<LOD	<LOD	0	0.1 [0.18]	0.03 [0.03, 0.55]	1
∑PFCA (C4–C14)	21.65 [11.72]	19.96 [3.25, 39.23]	11	29.16 [17.32]	21.27 [12.69, 69.09]	11	1.48 [0.68]	1.64 [0.55, 2.39]	7	3.42 [1.37]	3.36 [1.39, 5.93]	7
PFBS	1.88 [0.94]	1.74 [0.22, 3.5]	11	3.7 [5.07]	1.76 [0.09, 19]	11	0.05 [0.03]	0.05 [0.02, 0.12]	4	0.03 [0.03]	0.02 [0.02, 0.1]	1
PFPeS	0.24 [0.13]	0.23 [0.03, 0.42]	10	0.3 [0.12]	0.28 [0.18, 0.62]	11	0.05 [0.03]	0.03 [0.03, 0.09]	3	0.06 [0.05]	0.03 [0.03, 0.17]	3
L-PFHxS	0.94 [0.85]	0.61 [0.02, 2.59]	10	1.1 [0.66]	0.81 [0.41, 2.39]	11	0.03 [0.01]	0.02 [0.02, 0.04]	2	0.03 [0.02]	0.02 [0.02, 0.07]	2
Br-PFHxS	0.26 [0.23]	0.15 [0.01, 0.68]	9	0.27 [0.14]	0.26 [0.09, 0.57]	11	<LOD	<LOD	0	0.01 [0.01]	0.01 [0.01, 0.04]	2
∑PFHxS	1.2 [1.07]	0.78 [0.04, 3.27]	11	1.38 [0.78]	1.03 [0.55, 2.96]	11	0.03 [0.01]	0.02 [0.02, 0.06]	2	0.04 [0.04]	0.02 [0.02, 0.11]	2
PFHpS	0.07 [0.03]	0.06 [0.05, 0.13]	5	0.06 [0.03]	0.07 [0.03, 0.09]	7	<LOD	<LOD	0	<LOD	<LOD	0
Br-PFHpS	0.05 [0]	0.05 [0.05, 0.05]	2	0.03 [0.01]	0.03 [0.03, 0.05]	2	<LOD	<LOD	0	<LOD	<LOD	0
∑PFHpS	0.06 [0.04]	0.03 [0.03, 0.13]	5	0.07 [0.04]	0.07 [0.03, 0.14]	7	<LOD	<LOD	0	<LOD	<LOD	0
L-PFOS	0.8 [0.69]	0.61 [0.04, 2.09]	11	1.68 [0.86]	1.99 [0.46, 3.12]	11	0.07 [0.03]	0.06 [0.02, 0.12]	6	0.09 [0.07]	0.05 [0.02, 0.24]	5
Br-PFOS	0.9 [0.71]	0.75 [0.03, 2.22]	11	1.34 [0.57]	1.34 [0.42, 2.26]	11	0.06 [0.05]	0.06 [0.01, 0.17]	6	0.11 [0.09]	0.09 [0.01, 0.31]	6
∑PFOS	1.7 [1.38]	1.36 [0.06, 4.3]	11	3.02 [1.39]	3.59 [0.88, 5.38]	11	0.13 [0.08]	0.12 [0.02, 0.29]	6	0.19 [0.16]	0.16 [0.02, 0.54]	6
PFNS	<LOD	<LOD	0	<LOD	<LOD	0	<LOD	<LOD	0	<LOD	<LOD	0
PFDS	<LOD	<LOD	0	<LOD	<LOD	0	<LOD	<LOD	0	<LOD	<LOD	0
∑PFSA(C4–C10)	5.06 [3.16]	4.35 [0.81, 9.66]	11	8.49 [5.06]	7.76 [1.81, 22.39]	11	0.25 [0.12]	0.23 [0.14, 0.53]	7	0.33 [0.18]	0.28 [0.15, 0.75]	7
FBSA	0.11 [0.12]	0.03 [0.03, 0.32]	4	0.03 [0.03]	0.03 [0.03, 0.13]	1	<LOD	<LOD	0	<LOD	<LOD	0
FHxSA	<LOD	<LOD	0	<LOD	<LOD	0	<LOD	<LOD	0	<LOD	<LOD	0
FOSA	<LOD	<LOD	0	<LOD	<LOD	0	<LOD	<LOD	0	<LOD	<LOD	0
L-MeFOSAA	<LOD	<LOD	0	<LOD	<LOD	0	<LOD	<LOD	0	<LOD	<LOD	0
Br-MeFOSAA	<LOD	<LOD	0	0.02 [0.03]	0.01 [0.01, 0.11]	1	<LOD	<LOD	0	<LOD	<LOD	0
L-EtFOSAA	<LOD	<LOD	0	0.05 [0.09]	0.02 [0.02, 0.32]	2	<LOD	<LOD	0	<LOD	<LOD	0
Br-EtFOSAA	<LOD	<LOD	0	0.06 [0.14]	0.01 [0.01, 0.47]	2	<LOD	<LOD	0	0.01 [0.02]	0.01 [0.01, 0.06]	1
4:2 FTS	<LOD	<LOD	0	<LOD	<LOD	0	<LOD	<LOD	0	<LOD	<LOD	0
6:2 FTS	0.07 [0.06]	0.03 [0.03, 0.21]	5	0.34 [0.31]	0.26 [0.03, 1.19]	10	0.04 [0.02]	0.03 [0.03, 0.09]	2	0.11 [0.06]	0.14 [0.03, 0.19]	5
8:2 FTS	0.04 [0.04]	0.03 [0.03, 0.17]	1	0.21 [0.45]	0.03 [0.03, 1.58]	3	0.11 [0.08]	0.11 [0.03, 0.21]	4	0.39 [0.39]	0.21 [0.03, 1.03]	5
ADONA	<LOD	<LOD	0	0.04 [0.05]	0.03 [0.03, 0.2]	1	<LOD	<LOD	0	<LOD	<LOD	0
9Cl-PF3ONS	<LOD	<LOD	0	<LOD	<LOD	0	<LOD	<LOD	0	<LOD	<LOD	0
11Cl-PF3OUDS	<LOD	<LOD	0	<LOD	<LOD	0	<LOD	<LOD	0	<LOD	<LOD	0
PF4OPeA	<LOD	<LOD	0	<LOD	<LOD	0	<LOD	<LOD	0	<LOD	<LOD	0
PF5OHxA	<LOD	<LOD	0	<LOD	<LOD	0	<LOD	<LOD	0	<LOD	<LOD	0
3-6-OPFHpA	<LOD	<LOD	0	<LOD	<LOD	0	<LOD	<LOD	0	<LOD	<LOD	0
PFEESA	<LOD	<LOD	0	<LOD	<LOD	0	<LOD	<LOD	0	<LOD	<LOD	0
10:2 FTS	0.05 [0.08]	0.03 [0.03, 0.31]	1	0.47 [1.41]	0.03 [0.03, 4.93]	2	0.27 [0.23]	0.32 [0.03, 0.66]	5	0.64 [0.74]	0.35 [0.03, 1.95]	5
PFECHS	0.4 [0.44]	0.18 [0.03, 1.4]	10	0.59 [0.45]	0.47 [0.14, 1.74]	11	0.03 [0.01]	0.03 [0.03, 0.05]	1	<LOD	<LOD	0
4:2 FTA	<LOD	<LOD	0	<LOD	<LOD	0	<LOD	<LOD	0	<LOD	<LOD	0
6:2 FTA	<LOD	<LOD	0	<LOD	<LOD	0	<LOD	<LOD	0	<LOD	<LOD	0
8:2 FTA	<LOD	<LOD	0	<LOD	<LOD	0	<LOD	<LOD	0	<LOD	<LOD	0
6:2 diPAP	<LOD	<LOD	0	<LOD	<LOD	0	<LOD	<LOD	0	<LOD	<LOD	0
6:6 PFPi	<LOD	<LOD	0	0.5 [0.77]	0.03 [0.03, 2.51]	4	<LOD	<LOD	0	0.64 [0.27]	0.69 [0.03, 0.84]	6
8:2 PAP	<LOD	<LOD	0	<LOD	<LOD	0	<LOD	<LOD	0	<LOD	<LOD	0
EtFOSA	<LOD	<LOD	0	0.11 [0.27]	0.03 [0.03, 0.97]	1	<LOD	<LOD	0	0.19 [0.21]	0.03 [0.03, 0.59]	3
MeFOSA	<LOD	<LOD	0	<LOD	<LOD	0	<LOD	<LOD	0	<LOD	<LOD	0
MeFOSE	<LOD	<LOD	0	<LOD	<LOD	0	<LOD	<LOD	0	<LOD	<LOD	0
EtFOSE	<LOD	<LOD	0	<LOD	<LOD	0	<LOD	<LOD	0	<LOD	<LOD	0
PFHO-DA	1.17 [2.75]	0.4 [0.03, 9.83]	7	2.77 [5.81]	0.35 [0.03, 19.03]	7	<LOD	<LOD	0	<LOD	<LOD	0
∑Prec	1.85 [2.66]	0.76 [0.19, 9.96]	11	5.2 [5.74]	2.46 [0.99, 19.94]	11	0.45 [0.3]	0.51 [0.1, 0.92]	6	1.97 [1.48]	1.63 [0.13, 4.43]	6
total PFAS	407.02 [310.23]	455.82 [48.19, 1146.66]	11	235 [141.87]	176.62 [144.04, 666.27]	11	254.3 [162.1]	144.78 [99.18, 486.5]	7	318.69 [158.56]	370.94 [93.55, 527.55]	7

a All concentration in ng/L.

However, ultrashort chain (C2–C3) PFAS (mainly TFA) are by far the most dominant class found in both raw and produced drinking water samples, with concentrations ranging from 3 to 1100 ng/L, with a relative mass contribution of 49–99%, Figure S4 (TFA 30–1100 ng/L, and PFPrA 1–66 ng/L). For ultrashort chain PFAS, no significant differences were found between surface water and groundwater sources, nor between source and produced drinking water (Figure 2). This could be explained by the very high solubility of ultrashort chain PFAS, their low sorption to soil, sediment, and sorbents applied in drinking water treatment, and their resistance to biological/chemical degradation, resulting in their ubiquitous presence.^25^ Similar high levels of the ultrashort chain were reported earlier in German drinking water.^44,45^ Similarly to TFA, another ultrashort chain trifluoromethanesulfonic acid (CF3SO3H) (not included in this study) is expected to be detected in drinking water at a similar prevalence to TFA but in a lower level, as was reported previously for European drinking water, surface water, and groundwater.^45,46^

Significantly lower levels of perfluoroalkyl carboxylic acids (PFCA), perfluoroalkyl sulfonic acid (PFSA), and precursors are found in groundwaters compared to surface waters in our study. Concentrations of PFCA ranged from 0.4 to 95.1 ng/L, while PFSA concentrations ranged from 0.1 to 25.5 ng/L and PFAS precursors occurred in a similar range, 0.05–19.8 ng/L (Figures 2, S4). Soil infiltration, retardation, and travel times may play a significant role in delaying or preventing the longer (≥C4) PFAS from entering groundwater.^47,48^ In earlier studies excluding ultrashort chain PFAS, the PFCA were found as the dominant class in surface waters^13,49^ as explained by a slightly higher sorption affinity of the sulfonate group than the carboxylate group.^47^

In all raw and produced drinking waters, TFA, PFBA, and PFOA were detected, and in the majority of raw and produced drinking waters, PFOS, PFPeA, PFHxA, PFHpA, PFBS, and/or PFPeS were also found. Tables 2 and S3 provide further details per specific PFAS and location. One PFAS precursor, perfluoro-ethyl-cyclo-hexane sulfonate (PFECHS), was detected in 10 out of 11 samples of drinking water produced from surface water (0.06–1.4 ng/L), while detected in only one drinking water produced from groundwater. PFECHS is an eight-carbon cyclic PFAS and has been used as a replacement for PFOS in various formulations. It was already reported to be used in aircraft hydraulic fluids as an erosion inhibitor in the late 1940s.^50^ Similar to other long-chain and legacy PFAS, PFECHS has been reported in environmental media around the world, including drinking water.^51^ Hexa-fluoro-propylene-oxide-dimer acid (HFPO-DA) was present in 7 out of 11 drinking water produced from surface water (Table S3). HFPO-DA was observed in one sample (location 10) with a high level (9.8 ng/L) as a result of the contamination by the fluorochemical plant in the Netherlands.^35^

Although PFOS and PFOA have relatively recently been banned by an international restriction on use and production,^52,53^ they are still present in drinking water (PFOS 0.05–4.3 ng/L; PFOA 0.04–8.6 ng/L) (Table S3). Further curative measures such as remediation of these sources, use of alternative sources, or additional treatment would be needed to further diminish human exposure to these restricted compounds.

Despite the fact that only 56 PFAS were investigated in this study, more unknown PFAS are expected to be present in raw and drinking water. Determining the unknown PFAS can be achieved using other comprehensive analysis techniques additional to the targeted analysis such as non-target analysis, suspect screening, total organic fluorine, extractable organic fluorine measurements, and total oxidizable precursor assay approaches.^54−57^

### 3.3 PFAS Removal in Drinking Water Treatment

**Figure 3:**
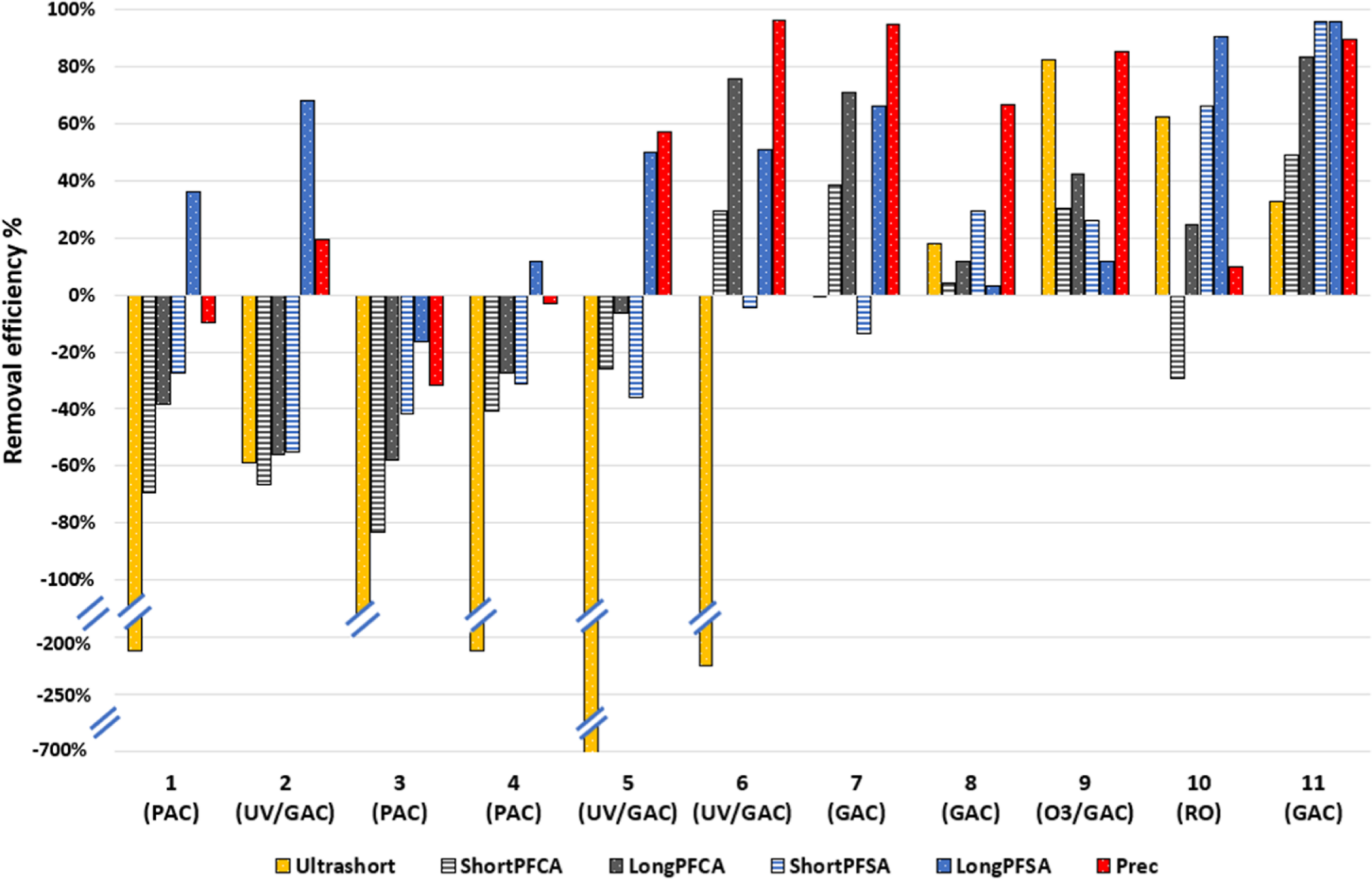
Removal efficiencies (%) in drinking water originated from surface water treated using advanced methods (GAC, PAC, UV/GAC, ozone/GAC, or RO) for each PFAS class: ultrashort (C2–C3), shortPFCA (C4–C6), longPFCA (C7–C14), shortPFSA (C4–C6), longPFSA (C7–C10), and Prec: a variety of precursors (C4–C24).

Removal efficiencies for PFAS during drinking water production using advanced treatment (GAC, PAC, RO, UV/GAC, or ozone/GAC) (Table 1) vary per sampling location, even when the same treatment process is being used (Figure 3). Negative removal (in particular for ultrashort chain) in locations 1–6, all using PAC or UV/GAC, might indicate breakthrough, desorption of earlier sorbed PFAS, degradation of PFAS precursors, and/or that materials used during treatment might leach PFAS. Desorption can be attributed to competition with other sorbing compounds (e.g., organic matter) leading over time to desorption and release of previously sorbed PFAS.^37,58^ The discrepancy between the different locations (sometimes with the same treatment process) could be explained by using different treatment setups for granular and powdered activated carbon, as well as by different sorbent ages. However, detailed information about the treatment setup and the age, type, and contact time of the sorbent were not available for all locations, so this cannot be further elaborated upon. Similarly, a full-scale study carried out in the US^59^ found a high breakthrough in treatment using GAC.

PFAS removal efficiencies also clearly vary in function of the chain length, where longer chain lengths in general show a better removal (Figure S5), which is in line with the literature.^60^ The short and ultrashort chain are less effectively removed by drinking water treatment due to their high mobility.^25,61^

### 3.4 Linear and Branched Isomers

**Figure 4:**
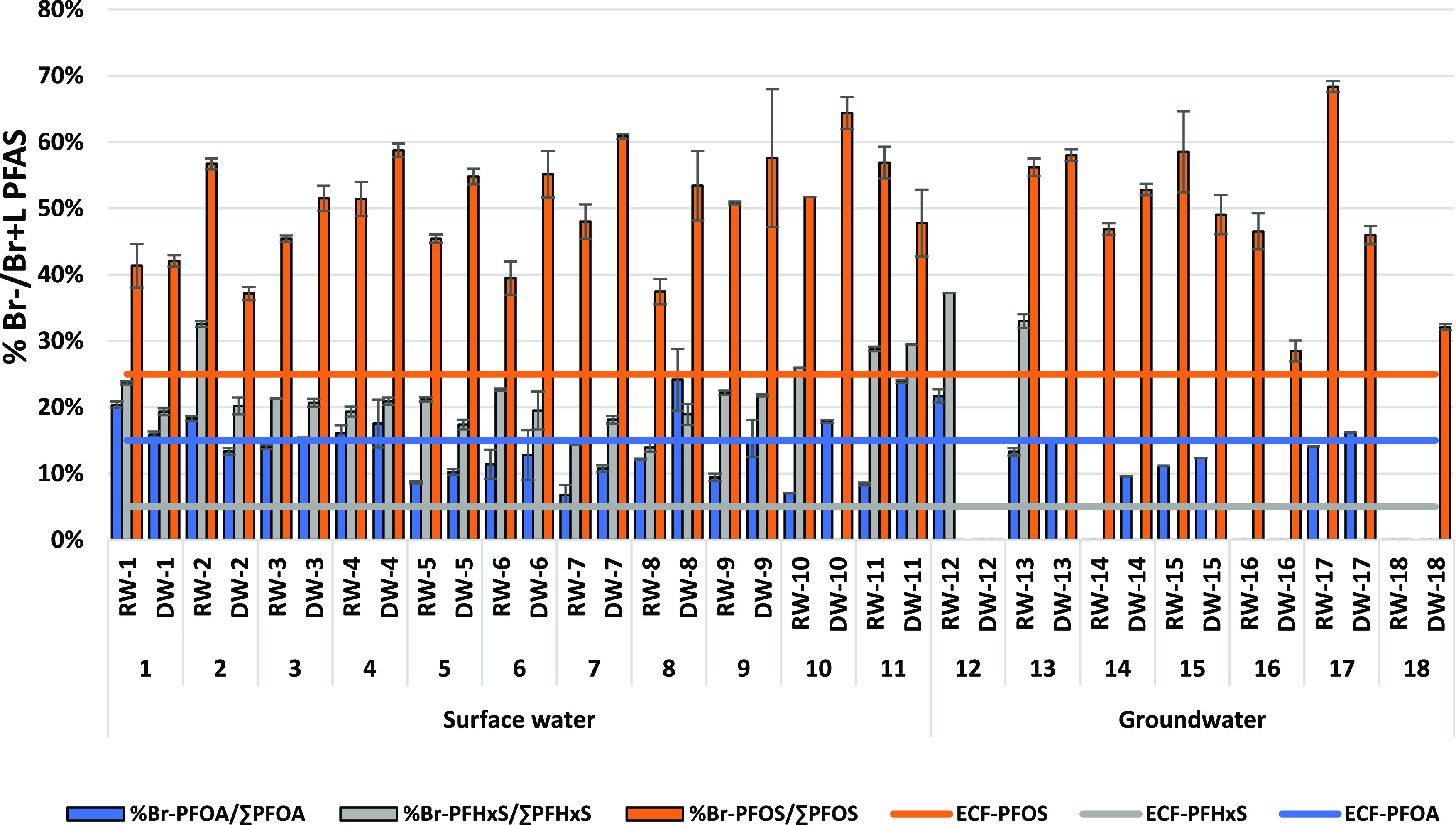
Relative contribution of the branched isomers for raw and produced drinking water. The contribution of the ECF production process is represented by a horizontal line for each PFAS. Error bar indicates the standard deviation from triplicate samples.

For PFOA, PFHpS, PFHxS, PFOS, MeFOSAA, and EtFOSAA, both branched (Br-) and linear (L-) isomers were analyzed (Table S1). Br-MeFOSAA and Br-EtFOSAA were not detected in any of the samples. Br-PFHxS was detected in raw and produced drinking water from surface water, but not in raw and produced drinking water from groundwater. Figure 4 shows the contribution of the branched isomers in raw and produced drinking water for PFOA, PFOS, and PFHxS. The contribution of branched isomers to their non-branched counterparts for both raw and produced drinking water ranges from 7 to 24% for PFOA, 17–37% for PFHxS, and is the highest with 25–68% for PFOS. There was no statistically significant difference (*p* > 0.05) in the isomer contribution (for the investigated PFAS) between different sources and different treatments (Figure 4).

The ECF process used in 80–90% of total PFAS produced before 2002^19^ yields approximately 20–30% branched isomer forms for PFOS, ∼5% for PFHxS, and 15–20% for PFOA.^20^ Nowadays, telomerization, which yields almost 100% linear form, is mainly used for PFAS production,^21^ which further decreases the proportion of branched isomers in the total mixture of PFAS used. The branched isomer contribution exceeds the original production mixture in both raw and produced drinking water for the two PFSA (PFHxS and PFOS), but not for PFOA (Figure 4). This overrepresentation of branched isomers of PFSA as compared with PFCA could be explained by different sorption behaviors between the hydrophilic functional group in both PFSA and PFCA isomers.^47^ Linear PFAS are more easily removed by treatment processes and environmental sorption processes due to the lower polarity and different sorption behavior of the linear isomer as compared to its branched counterpart.^58,62^ A slight but not significant increase of the branched isomer contribution (for PFCA and PFSA) in produced versus raw water was observed (Figure 4). The higher level of branched isomers present in drinking water as compared to the originally produced mixture indicates the need for further research on isomer-specific toxicity. Hardly any information on comparing hazards for branched versus linear PFAS isomers is available in the current literature, and what is available is based on associations and shows contradictory conclusions.^63−65^

### 3.5 Risk to Human Health Based on EFSA Scientific Opinion

**Figure 5:**
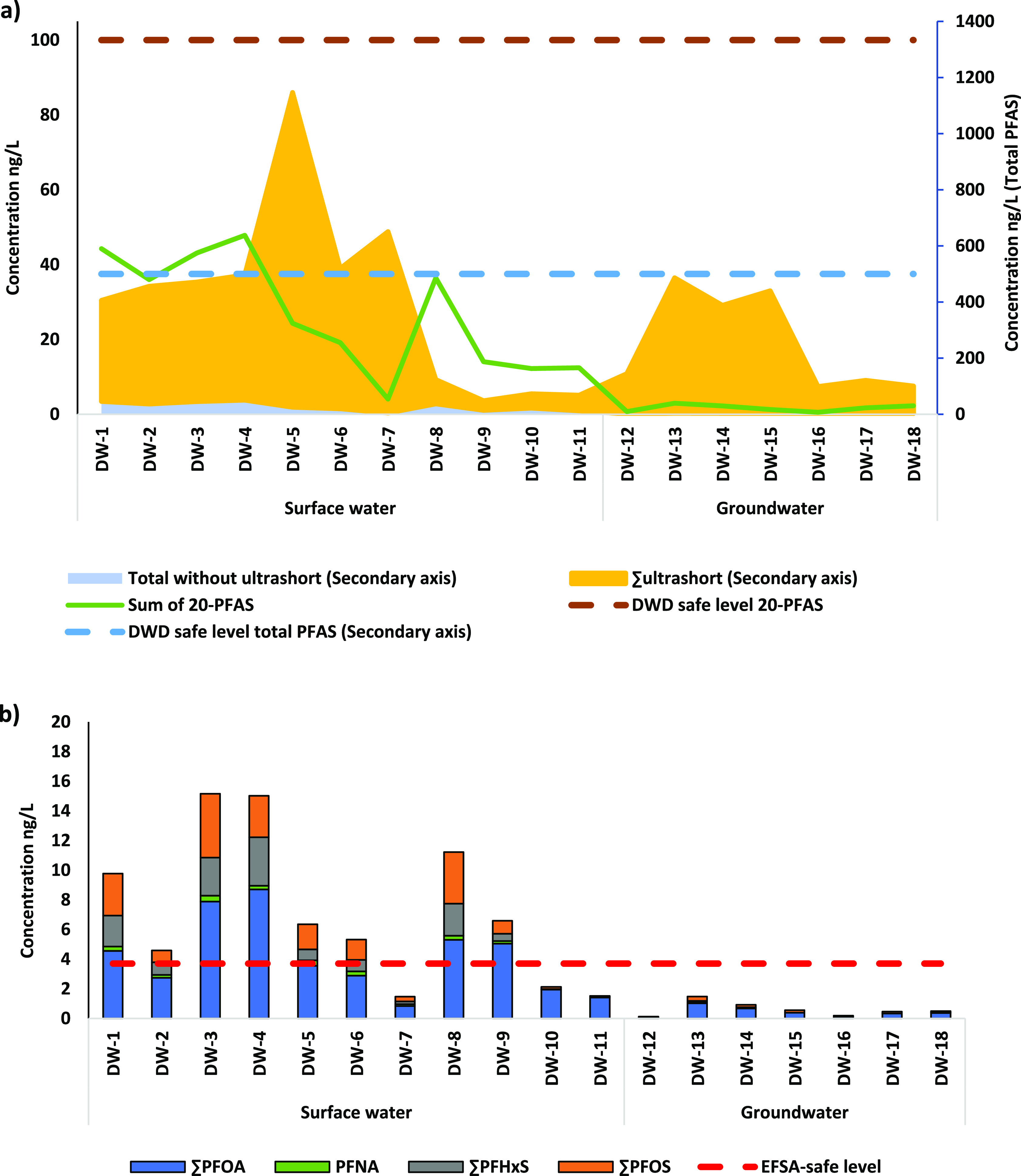
Occurrence of PFAS as compared to (a) binding DWD guidelines and (b) non-binding preliminary quality guideline. The red (b), brown [primary *y*-axis on the left, (b)], and light-blue [secondary *y*-axis on the right, (b)] dashed lines represent the safe level based on the non-binding EFSA 2020 (4-PFAS), DWD (20-PFAS), and DWD total PFAS, respectively. The green line [primary axis, to be compared with the brown line, figure (a)], light blue, and yellow shadows [secondary axis, to be compared with the dashed blue line, (a)] present, respectively, the concentration of the sum of 20 DWD-PFAS, total investigated PFAS without the ultrashort chain, and the ultrashort chain PFAS.

To assess possible adverse human health effects due to exposure to PFAS via drinking water, the occurrence of PFAS was compared to both the recently introduced binding DWD drinking water quality guidelines of 100 ng/L for 20 defined PFAS and/or 500 ng/L for total PFAS, as well as to the non-binding 3.7 ng/L preliminary guideline value for the four EFSA-PFAS (PFOS, PFOA, PFHxS, and PFNA, EFSA 2020) corresponding to the EFSA TWI of 4.4 ng/kg bodyweight (Figure 5).

None of the drinking water samples from our study exceeds the DWD value of 100 ng/L for the sum of 20 PFAS. However, 3 out of 11 drinking waters produced from surface water do exceed the DWD drinking water quality guideline of 500 ng/L for total PFAS, which is mainly explained by the high occurrence of ultrashort-chain PFAS (specifically TFA) that was included in the calculation. It has to be noted that, according to Annex 1 part B of the legislation, total PFAS means the totality of PFAS, which will only be applicable once technical guidelines for monitoring this parameter are developed. This is currently not the case. Furthermore, EU Member States may decide to use either one or both of the PFAS guidelines.

Only 3 out of 11 drinking waters produced from surface water, and all drinking waters produced from groundwater, meet the non-binding preliminary guideline value based on EFSA (2020). Similarly, Gebbink and van der Aa^35,66^ found a high level of four EFSA-PFAS in drinking water produced from surface water. Because ∼40% of the drinking water in the Netherlands originates from surface water, this demands attention for further source protection and further treatment where needed. The large contribution of the total PFAS (Figure 5) comes from the ultrashort PFAS (mainly TFA). Next to the development of guidelines for monitoring the sum PFAS parameter under the DWD, ultrashort PFAS (specially TFA) also need further research to evaluate if they pose risks to human health.

Most of the existing guidelines and regulations focus on PFOA and PFOS.^67,68^ The latest recommendation from the EFSA included four different PFAS to be considered, ignoring risks from other PFAS because of scarce toxicological data. Therefore, indicatively we also performed a preliminary risk assessment based on different RPFs proposed^32,33,39^ (Table 3). In the assessments, including 21-PFAS for Bil et al.,^32^ 7-PFAS for Bil et al.,^39^ and 7-PFAS for Rietjens et al.,^33^ thus not incorporating ultrashort PFAS, all drinking waters produced from groundwater have PEQ lower than the EFSA safe level 3.7 ng/L. On the contrary, drinking water samples produced from surface water have PEQ lower than 3.7 ng/L in only 2 out of 11 cases using RPFs based on both Bil et al. 2021, 2022, and 3 out of 11 when based on Rietjens et al. 2022 (Table 3). The EFSA guideline (2020) and other assessments did not explicitly mention any distinction between linear and branched isomers in their risk assessment. In this study, to provide a comprehensive assessment of the overall risk from PFAS isomers, linear and branched isomers were considered as one group in the evaluation, by assuming that linear and branched isomers have similar RPFs. Future research should consider the toxicity of branched isomers and ultrashort-chain PFAS and their risk for humans. However, following the recent EFSA opinion and mixture exposure (RPF) risk assessment, effective drinking water treatment techniques such as nanofiltration or RO will be needed to ensure the safety for drinking waters produced from surface water.

**Table 3:** Sum of PEQ Based on the RPF Results Per Individual Location in Produced DW

source type	sample code	sum 21-PFAS-PEQ (ng/L)^32^	sum 7-PFAS-PEQ (ng/L)^39^	sum 7-PFAS-PEQ (ng/L)^33^
surface water	DW-1	15.9 ≤ PEQ ≤ 23	17.1	10.3
	DW-2	7.9 ≤ PEQ ≤ 11.2	7.5	4.6
	DW-3	23.9 ≤ PEQ ≤ 31,1	26.2	16.5
	DW-4	20.3 ≤ PEQ ≤ 26.8	21.8	14.5
	DW-5	12.1 ≤ PEQ ≤ 16.1	12.4	7.4
	DW-6	10.1 ≤ PEQ ≤ 13.1	10.1	6.0
	DW-7	3.7 ≤ PEQ ≤ 5.1	3.9	2.1
	DW-8	17.7 ≤ PEQ ≤ 21	19.2	11.9
	DW-9	9.1 ≤ PEQ ≤ 9.9	9.1	6.8
	DW-10	3.1 ≤ PEQ ≤ 3.6	2.4	2.2
	DW-11	2 ≤ PEQ ≤ 2.4	1.6	1.5
groundwater	DW-12	0.1 ≤ PEQ ≤ 0.2	0.1	0.1
	DW-13	2.9 ≤ PEQ ≤ 3.7	3	1.8
	DW-14	2.9 ≤ PEQ ≤ 4.8	3.3	1.8
	DW-15	1.4 ≤ PEQ ≤ 2.4	1.7	1.1
	DW-16	0.3 ≤ PEQ ≤ 0.3	0.3	0.2
	DW-17	0.7 ≤ PEQ ≤ 0.7	0.7	0.5
	DW-18	1.6 ≤ PEQ ≤ 2.5	1.7	0.9

## Supporting Information Available

Quantification and quality control, scheme of analytical experiment design, recoveries of mass-labeled standard, response of mass-labeled standard, relative contribution of each PFAS class, list of target analytes and LOD, RPFs for specific PFAS, and concentration of PFAS in each sampling location (PDF)
