# Supplementary material for: Occurrence, Fate, and Related Health Risks of PFAS in Raw and Produced Drinking Water
Source: Environ Sci Technol. 2023 Feb 13;57(8):3062–74. doi: 10.1021/acs.est.2c06015 (PMC9979608; doi:10.1021/acs.est.2c06015)
Supplement: Supplementary file 1 — es2c06015_si_001.pdf [file es2c06015_si_001.pdf]

Supplementary information

Occurrence, fate and related health risks of PFAS in raw and produced drinking water

Mohammad Sadia[a*], Ingeborg Nollen[a], Rick Helmus[a], Thomas L. ter Laak[a,b], Frederic Béen[b], Antonia Praetorius[a], Annemarie P. van Wezel[a]

[a]Institute for Biodiversity and Ecosystem Dynamics, University of Amsterdam, P.O. Box 94240, 1090 GE Amsterdam, The Netherlands.

[b]KWR Water Research Institute, P.O. Box 1072, 3430 BB, Nieuwegein, The Netherlands

Corresponding author: email m.s.sadia@uva.nl

Summary: 18 pages, 1 section, 5 figures, 3 tables

The following information is included

Content		Page
Section 1	Quantification and quality control.	S2
Figure S1	Schematic of the experimental study design.	S3
Figure S2	Average recovery of mass-labeled standard in all water samples.	S3
Figure S3	Response of mass labeled standard in the drinking water extracted with different sorbent size.	S4
Figure S4	Relative contribution per PFAS class.	S4
Figure S5	Removal efficiencies (%) in drinking water regarding chain length (C4-C10)	S5
Table S1	Information about the analytical standards, and the method limit of detection (LOD).	S5
Table S2	Relative potency factors (RPF) for PFAS.	S9
Table S3	Results per individual PFAS and location	S10
Reference		S18

Quantification and quality control

Identification and confirmation of target compounds were achieved by: firstly accurate mass measurements of the protonated and deprotonated molecular ions within a mass window of 2 ppm; secondly retention time match (≤ 0.20 min) of analytes detected in samples with corresponding standards in calibration solution; thirdly match between one fragment or product ions of analytes acquired previously identified in calibration standard solution and those determined in samples. Thus, the acquisition of a high-resolution precursor ion in combination with at least one product ion and the LC relative retention time met more than the criteria requirement of 5 identification points for the HR-MSMS instrumentation. List of all target analytes, their exact mass, and product ion used for confirmation are shown in Table S1.

The branched isomers for (PFOS, PFHxS, EtFOSAA, MeFOSAA) were investigated and used branched isomers standard for quantification. For PFOA and PFHpS were investigated using the linear standard for the quantification (semi-quantification). No branched isomers were detected for other PFAS. All branched isomers were reported as the sum of all branched isomers for each of 6 PFAS.

Each batch of samples, three procedural blanks (Milli-Q water) were extracted simultaneously and analysed for assessing the background contamination introduced and originating throughout the extraction from various sources in the laboratory. One quality control sample (Milli-Q water spiked with native standards) was also extracted and analysed in order to simultaneously evaluate the repeatability of the analytical method and investigate any systematic errors. Quantification of the targeted analytes was based on internal calibration with its corresponding mass-labeled standard. For compounds that have no corresponding labelled analog standard, a mass-labeled standard with similar function group and contiguous carbon-chain length was used (Table S1). Methanol injections were carried out after and before the standard injection to assess any carryover or contamination in the LC system.

An isolator column (Waters Corporation Milford, USA) had been installed after the solvent mixer of the LC pump before the sample injector to separate any contamination associated/originating from the LC system.

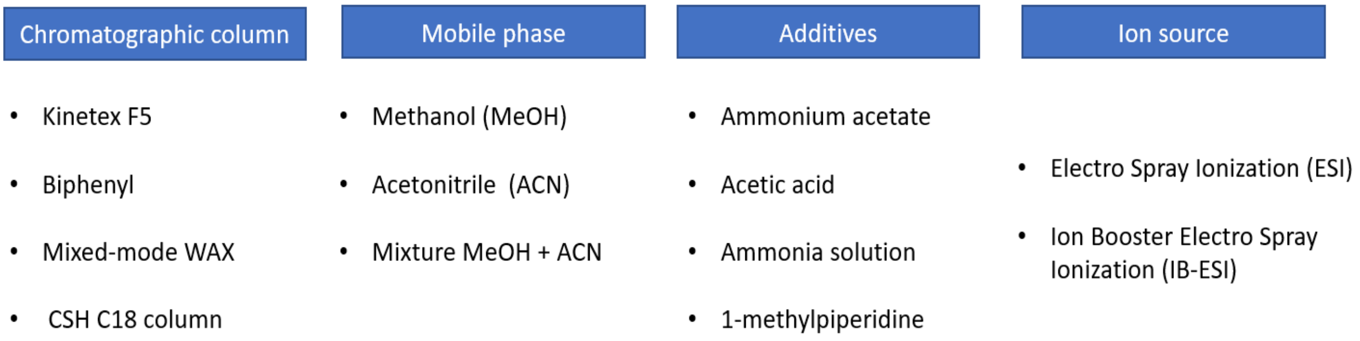

Figure S1. Schematic of the experimental study design for chromatographic and ionization optimization

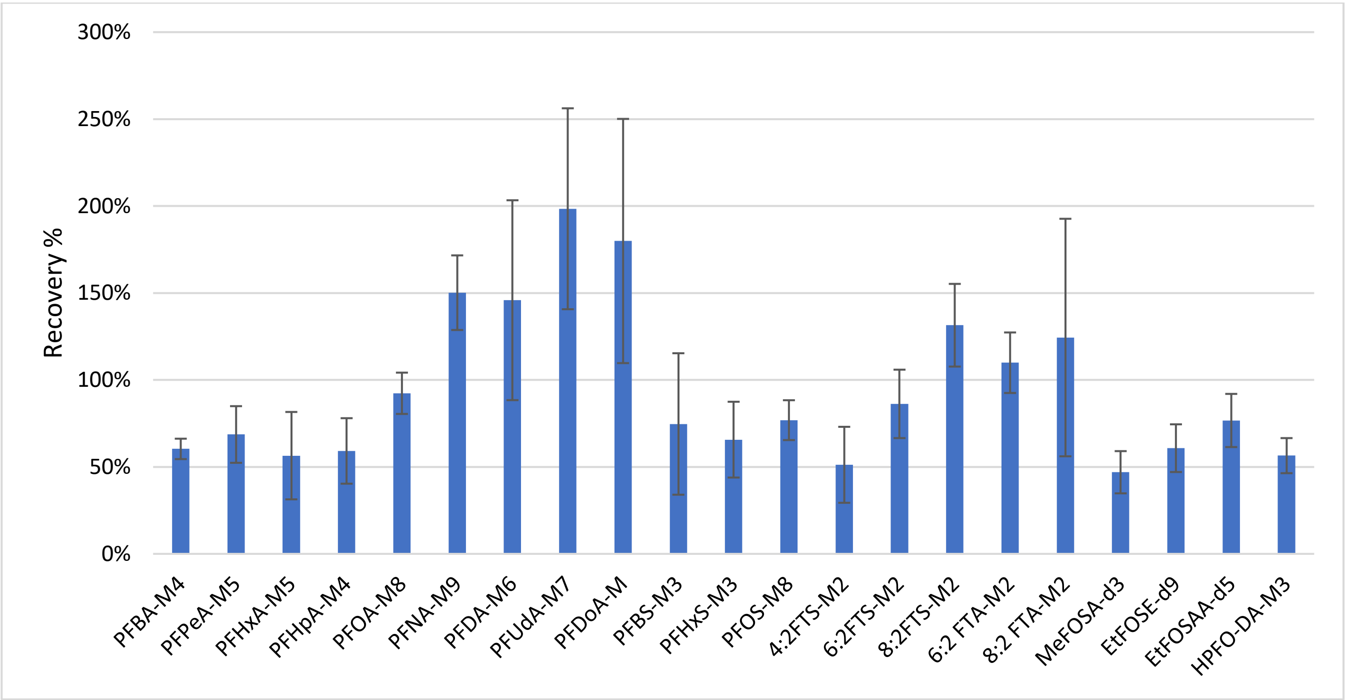

Figure S2. Average recoveries of mass-labeled standard in all water samples. Error bar indicates the standard deviation.

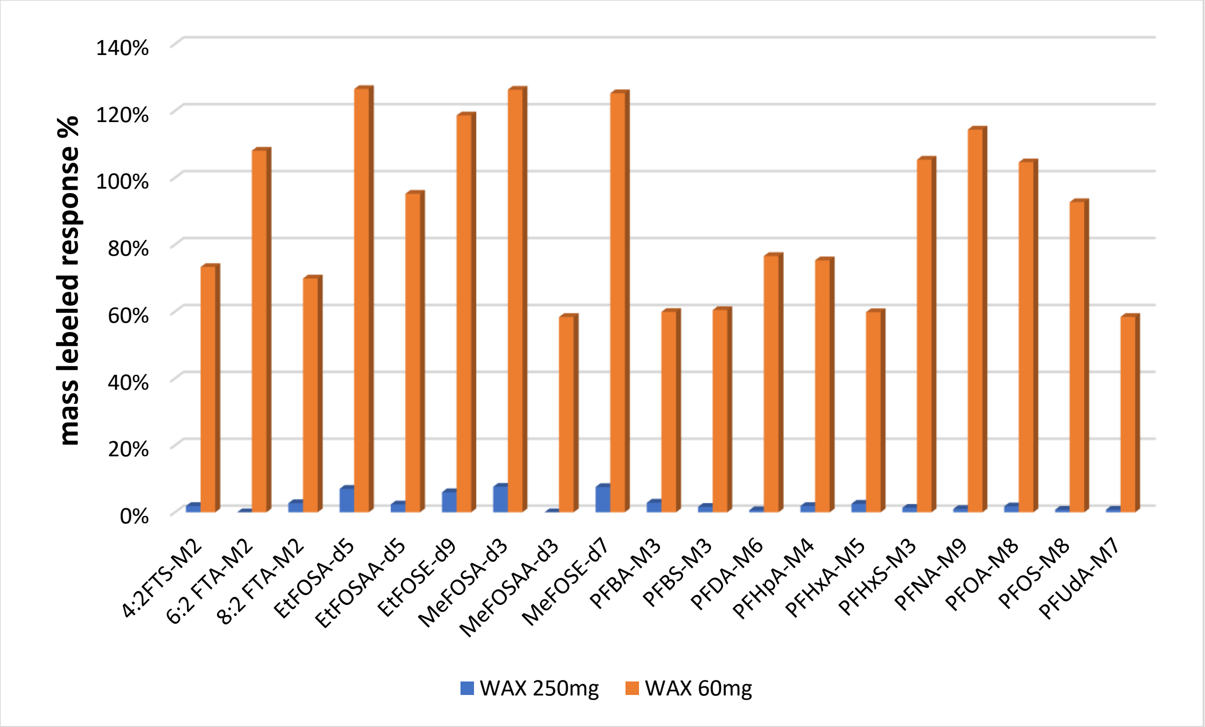

Figure S3. The response of 1 ng mass labeled standard in the drinking water extract extracted with different sorbent sizes used during extraction (weak anion exchange; WAX) 250 mg and 60 mg, as compared with pure solvent

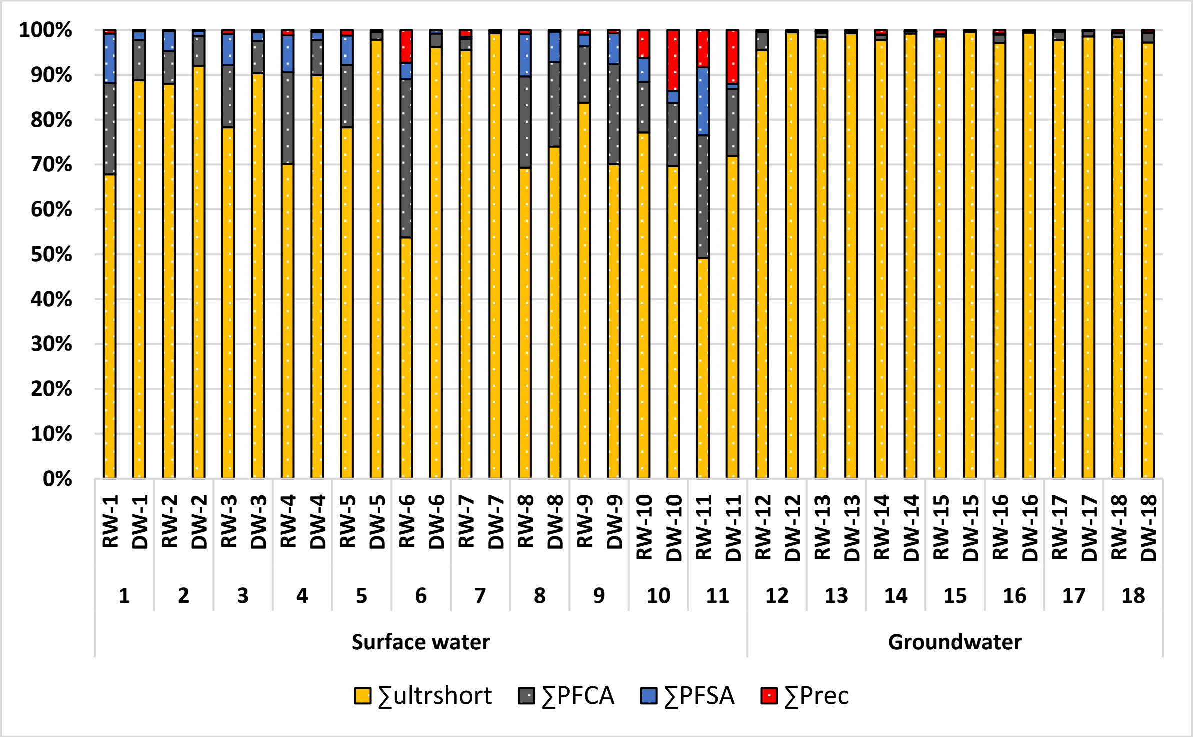

Figure S4. Relative contribution per PFAS class. PFAS class: ultrashort chain PFAS (C2-C3), PFCA: perfluorocarboxylic acids (C4-C14) PFSA: perfluorosulfonic acids (C4-C10), Prec: variety of precursors (C4-C24**)**

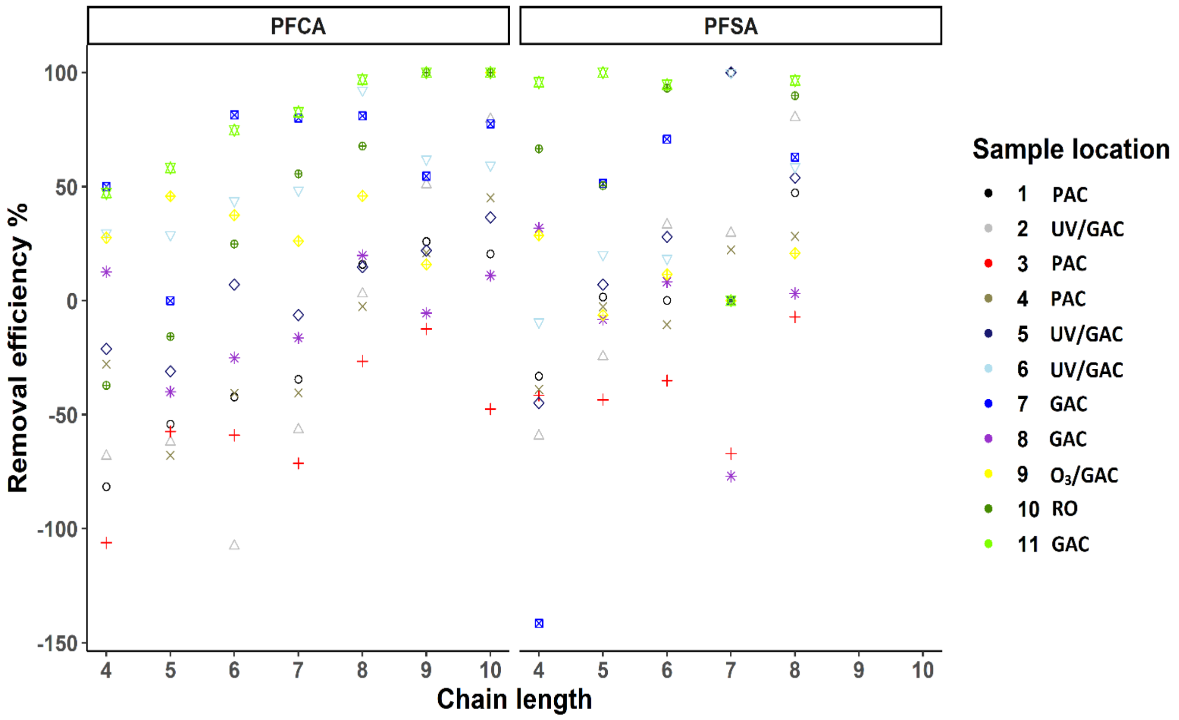

Figure S5. Removal efficiencies (%) in drinking water originated from surface water treated using advanced method (GAC, PAC, UV/GAC, Ozone/GAC, or RO) for chain length (C4-C10) for perfluoro-carboxylic acids (PFCA) and perfluoro-sulfonic acids (PFSA).

Table S1. Information about the analytical standards, and the method limit of detection (LOD).

PFAS class	*Compound*	*Acronym*	*Formula*	*Exact mass*	*Mass-labeled*	*Product ion*	*LOD (ng/L)*	*Accuracy*
Ultashort chain	Trifluoroacetic acid	TFA	$C_2HF_3O_2$	113,992867	M4PFBA	CF3	35.44	78%
	Pentafluoropropionic acid	PFPrA	$C_3HF_5O_2$	163,98967	M4PFBA	C2F5	0.23	80%
	Perfluoropropanesulfonic acid	PFPrS	$C_3HF_7O_3S$	249,953463	M3PFBS	SO3	0.04	114%
	Potassium pentafluoroethanesulfonate	PFEtS	$C_2HF_5O_3S$	199,95665	M3PFBS	SO3; FSO3	0.04	112%
perfluorocarboxylic acids	Perfluorobutyric acid	PFBA	$C_4HF_7O_2$	213,986476	M4PFBA	C3F7	0.05	110%
	Perfluoropentanoic acid	PFPeA	$C_5HF_9O_2$	263,983276	M5PFPeA	C4F9	0.05	95%
	Perfluorohexanoic acid	PFHxA	$C_6HF_{11}O_2$	313,980103	M5PFHxA	C5F11	0.05	105%
	Perfluoroheptanoic acid	PFHpA	$C_7HF_{13}O_2$	363,976898	M4PFHpA	C6F13	0.05	101%
	Perfluorooctanoic acid	L-PFOA	$C_8HF_{15}O_2$	413,973694	M8PFOA	C8F15O2	0.05	92%
		Br-PFOA	$C_8HF_{15}O_3$	413,973694	M8PFOA	C8F15O2	0.05	-
	Perfluorononanoic acid	PFNA	$C_9HF_{17}O_2$	463,97052	M9PFNA	C8F17	0.05	93%
	Perfluorodecanoic acid	PFDA	$C_{10}HF_{19}O2$	497,972412	M6PFDA	C9F19	0.05	90%
	Perfluoroundecanoic acid	PFUdA	$C_{11}HF_{21}O_2$	563,964111	M7PFUdA	C10F21	0.05	83%

	Perfluorododecanoic acid	PFDoA	$C_{12}HF_{23}O_2$	613,960938	MPFDoA	C12F23O2	0.05	86%
	Perfluorotridecanoic acid	PFTrDA	$C_{13}HF_{25}O_2$	663,957764	M7PFUdA	C13F25O2	0.05	82%
	Perfluorotetradecanoic acid	PFTeDA	$C_{14}HF_{27}O_2$	713,954529	M7PFUdA	C14F27O2	0.05	102%
perfluorosulfonic acids	Potassium perfluoro-1-butanesulfonate	PFBS	$C_4HF_9O_3S$	299,,950256	M3PFBS	FSO3;SO3	44	82%
	Sodium perfluoro-1-pentanesulfonate	PFPeS	$C_5HF_{11}O_3S$	349,947083	M3PFBS	FSO3;SO3	47	91%
	Potassium perfluorohexanesulfonate	L-PFHxS	$C_6HF_{13}O_3S$	399,943878	M3PFHxS	FSO3;SO3	37	105%
		Br-PFHxS	$C_6HF_{13}O_3S$	399,943878	M3PFHxS	FSO3;SO3	9	108%
	Sodium perfluoro-1-heptanesulfonate	L-PFHpS	$C_7HF_{15}O_3S$	449,940674	M3PFHxS	FSO3;SO3	0.05	96%
		Br-PFHpS	$C_7HF_{15}O_3S$	449,940674	M3PFHxS	FSO3;SO3	0.05	-
	Potassium perfluorooctanesulfonate	L-PFOS	$C_8HF_{17}O_3S$	499,9375	M8PFOS	FSO3;SO3	0.04	108%
		Br-PFOS	$C_8HF_{17}O_3S$	499,9375	M8PFOS	FSO3;SO3	0.01	105%
	Sodium perfluoro-1-nonanesulfonate	PFNS	$C_9HF_{19}O_3S$	549,934326	M8PFOS	FSO3;SO3	0.05	99%
	Sodium perfluoro-1-decanesulfonate	PFDS	$C_{10}HF_{21}O_3S$	599,931091	M8PFOS	FSO3;SO3	0.05	93%
Precursors	Perfluorobutylsulphonamide	FBSA	$C_4H_2F_9NO_2S$	298,966248	M3PFBS	NO2S	0.05	70%
	Perfluorohexanesulfonamide	FHxSA	$C_6H_2F_{13}NO_2S$	398,959869	M3PFHxS	NO2S	0.05	75%
	Perfluorooctanesulfonamide	FOSA	$C_8H_2F_{17}NO_2S$	498,953491	M8PFOS	NO2S	0.05	85%
	2,3,3,3-Tetrafluoro-2-(1,1,2,2,3,3,3-heptafluoropropoxy)propanoic acid	HFPO-DA	$C_6HF_{11}O_3$	329,975006	M3HFPO-DA	C3F7	0.05	98%
	N-methylperfluorooctane sulfonamidoacetic acid	L-MeFOSAA	$C_{11}H_6F_{17}NO_4S$	570,974609	d5-N-EtFOSAA	C8F17	0.04	93%
		Br-MeFOSAA	$C_{11}H_6F_{17}NO_4S$	570,974609	d5-N-EtFOSAA	C8F17	0.02	84%
	N-ethylperfluorooctane sulfonamidoacetic acid	L-EtFOSAA	$C_{12}H_8F_{17}NO_4S$	584,990234	d5-N-EtFOSAA	C8F17	0.04	95%
		Br-EtFOSAA	$C_{12}H_8F_{17}NO_4S$	584,990234	d5-N-EtFOSAA	C8F17	0.01	93%
	Sodium 1H, 1 H,2H,2H-perfluoro-1-hexanesulfonate	4:2FTS	$C_6H_5F_9O_3S$	327,981567	M2-4:2FTS	C5H3F6	0.05	107%
	Sodium 1 H, 1 H,2H,2H-perfluoro-1-octanesulfonate	6:2FTS	$C_8H_5F_{13}O_3S$	427,975189	M2-6:2FTS	C7HF8;C7F7	0.05	98%
	Sodium 1 H, 1 H,2H,2H-perfluoro-1-decanesulfonate	8:2FTS	$C_{10}H_5F_{17}O_3S$	527,968811	M2-8:2FTS	C9HF12	0.05	90%
	Sodium dodecafluoro-3H-4,8-dioxanonanoate	ADONA	$C_7H_2F_{12}O_4$	376,968322	M8PFOA	C4F9O2	0.05	100%
	Potassium 9-chlorohexadecafluoro-3-oxanonane-1-sulfonate	9Cl-PF3ONS	$C_8HClF_{16}O_4S$	531,902858	M8PFOA	C8F12ClO	0.05	92%

Potassium 11-chloroeicosafluoro-3-oxaundecane-1-sulfonate	11Cl-PF3OUDS	$C_{10}ClF_{20}HO_4S$	631,896471	M8PFOA	C8F20ClO	0.05	96%
Perfluoro-4-oxapentanoic acid	PF4OPeA	$C_4HF_7O_3$	229,981384	M4PFBA	CF3O	0.05	94%
Perfluro-5-oxahexanoic acid	PF5OHxA	$C_5HF_9O_3$	279,97821	M3PFHxS	CF3O	0.05	97%
Perfluoro-3,6-dioxaheptanoic acid	3-6-OPFHpA	$C_5HF_9O_4$	295,973114	M8PFOA	CF3O	0.05	88%
Potassium perfluoro(2-ethoxyethane)sulfonate	PFEESA	$C_4F_9HO_4S$	315,94519	M3PFHxS	CF3O	0.05	106%
Sodium 1 H, 1 H,2H,2H-perfluorododecanesulfonate	10:2FTS	$C_{12}H_5F_{21}O_3S$	627,962402	M2-8:2FTS	C12H3F20SO3	0.05	96%
N-(3-dimethylaminopropan-1-yl)perfluoro-1-hexanesulfonam ide	N-AP-FHxSA	$C_{11}H_{13}F_{13}N_2O_2S$	484,049011	M8PFOS		0.05	96%
N-(carboxymethyl)-N,N-dimethyl-N-[3-(1 H, 1 H ,2H ,2H-perfluoro-1-octanesulfonamido)propan-1-yl)ammonium	N-CMAmP-6:2FOSA	$C_{15}H_{19}F_{13}N_2O_4S$	570,085815	M8PFOS		0.05	97%
Potassium perfluoro-4-ethylcyclohexanesulfonate	PFECHS	$C_8HF_{15}O_3S$	461,.940674	M8PFOA	C8F15;FSO3	0.05	97%
3-Perfluoropropyl propanoic acid (3:3)	4:2FTA	$C_6H_5F_7O_2$	242,017776	M2-6:2FTA		0.05	97%
3-Perfluoropentyl propanoic acid (5:3)	6:2FTA	$C_8H_5F_{11}O_2$	342,011383	M2-6:2FTA		0.05	95%
3-Perfluoroheptyl propanoic acid (7:3)	8:2FTA	$C_{10}H_5F_{15}O_2$	442,005005	M2-8:2FTA		0.1	98%
Sodium bis(perfluorohexyl)phosphinate	6:6PFPi	$C_{12}HF_{26}O_2P$	701,929871	M5PFHxA	C6F14O2P	0.2	-
Sodium perfluorohexylperfluorooctylphosphinate	6:8PFPi	$C_{14}HF_{30}O_2P$	801,923523	M8PFOA		0.2	-
Sodium bis(perfluorooctyl)phosphinate	8:8PFPi	$C_{16}HF_{34}O_2P$	901,917114	M8PFOA		0.2	-
Sodium 1H,1H,2H,2H-perfluorooctylphosphate	6:2PAP	$C_8H_6F_{13}O_4P$	443,979614	M8PFOA	H2O4P	0.2	-
Sodium 1H,1H,2H,2H-perfluorodecylphosphate	8:2PAP	$C_{10}H_6F_{17}O_4P$	543,973206	M8PFOA	H2O4P	0.2	-
Sodium bis(1H,1H,2H,2H-perfluorooctyl)phosphate	6:2diPAP	$C_{16}H_9F_{26}O_4P$	789,9823	M8PFOA	H2O4P	0.2	72%
Sodium bis(1H,1H,2H,2H-perfluorodecyl)phosphate	8:2diPAP	$C_{20}H_9F_{34}O_4P$	989,969543	M8PFOA	C10H5F17O4P	0.2	-
N-ethylperfluoro-1-octanesulfonamidoacetic acid	N-EtFOSA	$C_{10}H_6F_{17}NO_2S$	526,984802	d9-N-EtFOSE	C3F7	0.05	104%
N-methylperfluoro-1-octanesulfonamidoacetic acid	N-MeFOSA	$C_9H_4F_{17}NO_2S$	512,.969116	d3-N-MeFOSA	C3F7	0.05	93%
2-(N-ethylperfluoro-1-octanesulfonamido)-ethanol	N-EtFOSE	$C_{12}H_{10}F_{17}NO_3S$	571,010986	d3-N-MeFOSA	C2H3O2	0.05	98%

	2-(N-methylperfluoro-1-octanesulfonamido)-ethanol	N-MeFOSE	$C_{11}H_8F_{17}NO_3S$	556,995361	d9-N-EtFOSE	C2H3O2	0.05	98%
	N-(methyl) nonafluoro-butanesulfonamide	MeFBSA	$C_5H_4F_9NO_2S$	312,981903	M3PFBS	NO3S	0.05	97%
Extraction standard	Perfluoro-n-[^{13}C4]butanoic acid	M4PFBA	13^$C_4HF_7O_2$	219,007173				
	Perfluoro-n-[^{13}C5]pentanoic acid	M5PFPeA	13^$C_5HF_9O_2$	270,007334				
	Perfluoro-n-[1,2,3,4,6-^{13}C5]hexanoic acid	M5PFHxA	13^$C_5CHF_{11}O_2$	320,00414				
	Perfluoro-n-[1,2,3,4-^{13}C4]heptanoic acid	M4PFHpA	13^$C_4C_3HF_{13}O_2$	368,997592				
	Perfluoro-n-[^{13}C8]octanoic acid	M8PFOA	13^$C_8HF_{15}O_2$	423,007818				
	Perfluoro-n-[^{13}C9]nonanoic acid	M9PFNA	13^$C_9HF_{17}O_2$	474,007979				
	Perfluoro-n-[1,2,3,4,5,6-^{13}C6]decanoic acid	M6PFDA	13^$C_6C_4HF_{19}O_2$	520,994721				
	Perfluoro-n-[1,2,3,4,5,6,7-^{13}C7]undecanoic acid	M7PFUdA	13^$C_7C_4HF_{21}O_2$	571,994882				
	Perfluoro-n-[1,2-^{13}C]dodecanoic acid	MPFDoA	13^$C_2C_{10}HF_{23}O_2$	616,974914				
	2,3,3,3-Tetrafluoro-2-(1, 1,2,2,3,3,3-heptafluoropropoxy)-^{13}C3-propanoic acid	M3HFPO-DA	13^$C_3C_3HF_{11}O_3$	333,992345				
	Sodium perfluoro-1-[2,3,4-^{13}C3]butanesulfonate	M3PFBS	13^$C_3CHF_9O_3S$	303,96761				
	Sodium perfluoro-1-[1,2,3-^{13}C3]hexanesulfonate	M3PFHxS	13^$C_3C_3H_2F_{13}O_3S$	404,969048				
	Sodium perfluoro-1-[^{13}C8]octanesulfonate	M8PFOS	13^$C_8HF_{17}O_3S$	508,97161				
	Sodium 1 H, 1 H,2H,2H-perfluoro-1-[1,2-^{13}C2]hexanesulfonate	M2-4:2FTS	13^$C_2C_4H_5F_9O_3S$	330,995555				
	Sodium 1 H, 1 H,2H,2H-perfluoro-1-[1,2-^{13}C2]octanesulfonate	M2-6:2FTS	13^$C_2C_6H_5F_{13}O_3S$	430,989168				
	Sodium 1 H, 1 H,2H,2H-perfluoro-1-[1,2-^{13}C2]decanesulfonate	M2-8:2FTS	13^$C_2C_8H_5F_{17}O_3S$	530,982781				
	2-Perfluorohexyl-[1,2-^{13}C2]-ethanoic acid	M2-6:2FTA	13^$C_2C_6H_3F_{13}O_2$	381,006532				
	2-Perfluorooctyl-[1,2-^{13}C2]-ethanoic acid	M2-8:2FTA	13^$C_2C_8H_3F_{17}O_2$	481,000145				
	2-Perfluorodecyl-[1,2-^{13}CJ-ethanoic acid	M2-10:2FTA	13^$C_2C_{10}H_3F_{21}O_2$	580,993758				
	Sodium bis(1 H, 1 H,2H,2H-[1,2-^{13}C2]perfluorooctyl)phosphate	M4-6:2diPAP	13^$C_4C_{12}H_9F_{26}O_4P$	795,003025				
	Sodium bîs(1 H, 1 H,2H,2H-[1,2-^{13}C2]perfluorodecyl)phosphate	M4-8:2diPAP	13^$C_4C_{16}H_9F_{34}O_4P$	994,990251				
	N-methyl-d_3 -perfluoro-1-octanesulfonamide	d_3-N-MeFOSA	2^$H_3C_9HF_{17}NO_2S$	516,995236				

	Name	Abbreviation	Formula	Mass
	2-(N-ethyl-d_5-perfluoro-1-octanesulfonamido)ethan-d_4-ol	d_9-N-EtFOSE	2^$H_9C_{12}HF_{17}NO_3S$	581,074761
	N-Ethyl-n-perfluorooctanesulfonamidoacetic acId-d_5	d_5-N-EtFOSAA	2^$H_5C_{12}H_3F_{17}NO_4S$	591,028919
Injection standard	Perfluoro-n-[2,3,4-^{13}C3]butanoic acid	M3PFBA	13^C_3CHF_7O2	218,003818
	Perfluoro-n-(1,2-^{13}C2]octanoic acid	M2PFOA	13^$C_2C_6HF_{15}O_2$	416,987689
	Sodium perfluoro-1-[1,2,3,4-^{13}C4]octanesulfonate	M4PFOS	13^$C_4C_4HF_{17}O_3S$	504,95819
	N-ethyl-d_5-perfluoro-1-octanesulfonamide	d_5-N-EtFOSA-M	^{2}^$H_5C_{10}HF_{17}NO_2S$	533,023439
	2-(N-methyl-d_3-perfluoro-1-octanesulfonamido)ethan-d_4-ol	d_7-N-MeFOSE-M	^{2}^$H_7C_{11}HF_{17}NO_3S$	565,046558
	N-Methyl-n-perfiuorooctanesu!fonamidoacetk acld-d_3	N-MeFOSAA-d_3	^{2}^$H_3C_{11}H_3F_{17}NO_4S$	575,000715

Table S2. Relative potency factors (RPF) for PFAS reported in the Bil et al and Rietjens et al studies and used to calculate the perfluorooctanoic acid equivalent (PEQ).

	RPF (BIL ET AL., 2021)	*RPF (BIL ET AL., 2022)*	*RPF (RIETJENS ET AL, 2021)*
FBS	0,001	0,023	0,008
PFPES	0,001≤ RPF ≥0,6	-	-
PFHXS	0,6	0,91	0,24
PFHPS	0,6 ≤ RPF ≥2	-	-
PFOS	2	2,6	1,5
PFDS	2	-	-
PFBA	0,05	-	-
PFPEA	0,01 ≤ RPF ≥0,05	-	-
PFHXA	0,01	0,011	0,003
PFHPA	0,01 ≤ RPF ≥ 1	-	-
PFOA	1	1	1
PFNA	10	7,2	1,5
PFDA	4 ≤ RPF ≥ 10	5,9	2,9
PFUNDA	4	-	-
PFDODA	3	-	-
PFTRDA	0,3 ≤ RPF ≥ 3	-	-
PFTEDA	0,3	-	-
PFHXDA	0,02	-	-
PFODA	0,02	-	-
HFPO-DA	0,06	-	-
ADONA	0,03	-	-

Table S3. Results per individual PFAS and location. The concentration in ngg/L. AV, and SD represent the average and standard deviation of the triplicate analysis, respectively.

Surface water

	1				2				3				4				5				6			
	DW-1		RW-1		DW-2		RW-2		DW-3		RW-3		DW-4		RW-4		DW-5		RW-5		DW-6		RW-6	
	AV	ST	AV	ST	AV	ST	AV	ST	AV	ST	AV	ST	AV	ST	AV	ST	AV	ST	AV	ST	AV	ST	AV	ST
TFA	296.24	10.26	106.07	0.49	377.42	26.64	263.88	3.34	359.86	17.56	154.60	1.33	381.44	19.11	133.90	1.47	1104.60	211.08	131.56	1.57	477.05	31.68	144.00	4.09
PFPrA	63.70	3.84	<LOD	0.00	41.52	1.45	<LOD	0.00	65.55	8.19	<LOD	0.00	64.45	12.15	<LOD	0.00	16.74	0.70	<LOD	0.00	22.22	4.96	1.04	0.14
PFPrS	0.17	0.01	0.13	0.00	0.20	0.00	0.09	0.00	0.16	0.01	0.11	0.01	0.16	0.00	0.11	0.00	0.08	0.01	0.10	0.00	0.06	0.00	0.07	0.00
PFEtS	<LOD	0.00	<LOD	0.00	<LOD	0.00	<LOD	0.00	<LOD	0.00	<LOD	0.00	<LOD	0.00	<LOD	0.00	<LOD	0.00	<LOD	0.00	<LOD	0.00	<LOD	0.00
∑ultrshort	360.11	0.00	106.20	0.00	419.14	0.00	263.97	0.00	425.57	0.00	154.71	0.00	446.04	0.00	134.01	0.00	1121.43	0.00	131.66	0.00	499.33	0.00	145.12	0.00
PFBA	10.18	0.57	5.61	0.56	13.41	0.30	7.98	1.40	8.05	0.21	3.90	0.18	10.82	1.20	8.47	0.46	4.35	0.32	3.59	0.09	3.60	0.31	5.12	0.44
PFPeA	6.89	0.39	4.47	0.11	3.96	0.03	2.44	0.18	5.44	0.13	3.46	0.06	6.65	0.40	3.97	0.18	4.27	0.24	3.26	0.09	3.31	0.27	4.66	0.32
PFHxA	8.80	1.64	6.19	0.34	7.60	0.01	3.66	0.19	6.85	0.34	4.31	0.20	7.61	0.37	5.41	0.19	4.62	0.06	4.97	0.15	3.34	0.19	5.94	0.11
PFHpA	5.68	0.62	4.22	0.06	2.59	0.05	1.66	0.03	4.74	0.23	2.76	0.06	5.05	0.22	3.59	0.13	2.63	0.09	2.47	0.06	2.01	0.06	3.90	0.29
L-PFOA	3.63	0.07	4.56	0.07	2.23	0.06	2.44	0.05	6.68	0.05	5.36	0.12	7.17	0.35	7.12	0.42	3.19	0.12	3.80	0.15	2.52	0.10	32.97	5.69
Br-PFOA	0.93	0.01	0.86	0.03	0.50	0.02	0.38	0.01	1.21	0.01	0.87	0.01	1.52	0.37	1.37	0.07	0.36	0.03	0.36	0.02	0.37	0.15	4.24	0.12
∑PFOA	4.55	0.00	5.42	0.00	2.73	0.00	2.82	0.00	7.89	0.00	6.23	0.00	8.70	0.00	8.48	0.00	3.55	0.00	4.17	0.00	2.89	0.00	37.21	0.00
PFNA	0.30	0.01	0.40	0.01	0.20	0.01	0.41	0.00	0.40	0.02	0.35	0.02	0.26	0.02	0.33	0.02	0.36	0.00	0.47	0.01	0.29	0.01	0.75	0.06
PFDA	0.17	0.02	0.22	0.00	0.05	0.00	0.24	0.00	0.30	0.00	0.21	0.01	0.15	0.01	0.27	0.02	0.18	0.02	0.28	0.01	0.14	0.01	0.34	0.12
PFUdA	<LOD	0.00	<LOD	0.00	<LOD	0.00	<LOD	0.00	<LOD	0.00	<LOD	0.00	<LOD	0.00	<LOD	0.00	<LOD	0.00	<LOD	0.00	0.07	0.00	<LOD	0.00
PFDoA	<LOD	0.00	<LOD	0.00	<LOD	0.00	<LOD	0.00	<LOD	0.00	<LOD	0.00	<LOD	0.00	<LOD	0.00	<LOD	0.00	<LOD	0.00	<LOD	0.00	<LOD	0.00
PFTrDA	<LOD	0.00	<LOD	0.00	<LOD	0.00	<LOD	0.00	<LOD	0.00	<LOD	0.00	<LOD	0.00	<LOD	0.00	<LOD	0.00	<LOD	0.00	<LOD	0.00	<LOD	0.00
PFTeDA	<LOD	0.00	<LOD	0.00	<LOD	0.00	<LOD	0.00	<LOD	0.00	<LOD	0.00	<LOD	0.00	<LOD	0.00	<LOD	0.00	<LOD	0.00	<LOD	0.00	<LOD	0.00
∑PFCA	36.57	0.00	31.93	0.00	30.55	0.00	22.03	0.00	33.66	0.00	27.45	0.00	39.23	0.00	38.99	0.00	19.96	0.00	23.37	0.00	15.65	0.00	95.13	0.00
PFBS	2.34	0.12	1.76	0.04	3.39	0.06	2.13	0.01	2.05	0.07	1.45	0.05	2.03	0.03	1.46	0.06	1.69	0.04	1.17	0.01	1.17	0.04	1.07	0.04
PFPeS	0.34	0.02	0.34	0.02	0.35	0.01	0.28	0.01	0.42	0.01	0.29	0.01	0.40	0.02	0.38	0.02	0.23	0.01	0.25	0.01	0.18	0.02	0.23	0.01
L-PFHxS	1.59	0.03	1.68	0.01	0.58	0.02	1.02	0.01	2.03	0.05	1.50	0.04	2.59	0.09	2.39	0.09	0.61	0.03	0.81	0.01	0.63	0.21	0.74	0.02
Br-PFHxS	0.49	0.01	0.40	0.01	0.28	0.01	0.26	0.01	0.53	0.01	0.41	0.01	0.68	0.01	0.57	0.03	0.13	0.01	0.22	0.00	0.15	0.08	0.22	0.01
∑PFHxS	2.08	0.00	2.08	0.00	0.85	0.00	1.28	0.00	2.57	0.00	1.90	0.00	3.27	0.00	2.96	0.00	0.74	0.00	1.03	0.00	0.78	0.00	0.96	0.00
PFHpS	<LOD	0.00	0.09	0.00	0.05	0.00	0.07	0.00	0.08	0.00	0.08	0.00	0.06	0.00	0.09	0.00	<LOD	0.00	0.07	0.00	<LOD	0.00	0.07	0.00
Br-PFHpS	<LOD	0.01	0.05	0.02	<LOD	0.00	<LOD	0.00	0.05	0.00	<LOD	0.01	0.05	0.01	0.05	0.01	<LOD	0.00	<LOD	0.00	<LOD	0.00	<LOD	0.01

∑PFHpS	<LOD	0.00	0.14	0.00	0.05	0.00	0.07	0.00	0.13	0.00	0.08	0.00	0.11	0.00	0.14	0.00	<LOD	0.00	0.07	0.00	<LOD	0.00	0.07	0.00
L-PFOS	1.66	0.15	3.12	0.00	0.35	0.03	2.57	0.00	2.09	0.03	2.19	0.00	1.15	0.05	1.89	0.00	0.76	0.06	2.00	0.00	0.61	0.01	1.99	0.00
Br-PFOS	1.17	0.09	2.26	0.05	0.46	0.02	1.52	0.06	2.22	0.16	1.83	0.09	1.64	0.05	2.00	0.19	0.93	0.05	1.67	0.10	0.75	0.09	1.30	0.49
∑PFOS	2.83	0.00	5.38	0.00	0.81	0.00	4.09	0.00	4.30	0.00	4.02	0.00	2.80	0.00	3.89	0.00	1.69	0.00	3.67	0.00	1.36	0.00	3.29	0.00
PFNS	<LOD	0.00	<LOD	0.00	<LOD	0.00	<LOD	0.00	<LOD	0.00	<LOD	0.00	<LOD	0.00	<LOD	0.00	<LOD	0.00	<LOD	0.06	<LOD	0.00	<LOD	0.00
PFDS	<LOD	0.00	<LOD	0.00	<LOD	0.00	<LOD	0.00	<LOD	0.00	<LOD	0.00	<LOD	0.00	<LOD	0.00	<LOD	0.00	<LOD	0.00	<LOD	0.00	<LOD	0.00
∑PFSA	7.67	0.00	17.31	0.00	5.45	0.00	13.34	0.00	9.46	0.00	13.80	0.00	8.60	0.00	15.83	0.00	4.37	0.00	11.02	0.00	3.49	0.00	9.97	0.00
FBSA	0.32	0.03	<LOD	0.00	0.22	0.00	<LOD	0.00	0.26	0.00	<LOD	0.00	0.24	0.01	<LOD	0.00	<LOD	0.00	<LOD	0.00	<LOD	0.00	0.13	0.03
FHxSA	<LOD	0.00	<LOD	0.00	<LOD	0.00	<LOD	0.00	<LOD	0.00	<LOD	0.00	<LOD	0.00	<LOD	0.00	<LOD	0.00	<LOD	0.00	<LOD	0.00	<LOD	0.00
FOSA	<LOD	0.00	<LOD	0.00	<LOD	0.00	<LOD	0.00	<LOD	0.00	<LOD	0.00	<LOD	0.00	<LOD	0.00	<LOD	0.00	<LOD	0.00	<LOD	0.00	<LOD	0.00
L-MeFOSAA	<LOD	0.00	<LOD	0.00	<LOD	0.00	<LOD	0.00	<LOD	0.00	<LOD	0.00	<LOD	0.00	<LOD	0.00	<LOD	0.00	<LOD	0.00	<LOD	0.00	<LOD	0.00
Br-MeFOSAA	<LOD	0.00	<LOD	0.00	<LOD	0.00	<LOD	0.00	<LOD	0.00	<LOD	0.00	<LOD	0.00	<LOD	0.00	<LOD	0.00	<LOD	0.00	<LOD	0.00	<LOD	0.00
L-EtFOSAA	<LOD	0.00	<LOD	0.00	<LOD	0.00	<LOD	0.00	<LOD	0.00	<LOD	0.00	<LOD	0.00	<LOD	0.00	<LOD	0.00	<LOD	0.00	<LOD	0.00	<LOD	0.00
Br-EtFOSAA	<LOD	0.00	<LOD	0.00	<LOD	0.00	<LOD	0.00	<LOD	0.00	<LOD	0.00	<LOD	0.00	<LOD	0.00	<LOD	0.00	<LOD	0.00	<LOD	0.00	<LOD	0.00
4:2FTS	<LOD	0.00	<LOD	0.00	<LOD	0.00	<LOD	0.00	<LOD	0.00	<LOD	0.00	<LOD	0.00	<LOD	0.00	<LOD	0.00	<LOD	0.00	<LOD	0.00	<LOD	0.00
6:2FTS	<LOD	0.02	0.28	0.03	0.17	0.01	0.31	0.01	0.09	0.03	0.15	0.02	0.05	0.02	0.19	0.01	0.21	0.00	1.19	0.04	0.12	0.02	0.16	0.02
8:2FTS	<LOD	0.00	<LOD	0.00	<LOD	0.00	<LOD	0.00	<LOD	0.00	<LOD	0.00	<LOD	0.00	<LOD	0.00	<LOD	0.00	<LOD	0.00	<LOD	0.00	<LOD	0.00
ADONA	<LOD	0.00	<LOD	0.00	<LOD	0.00	<LOD	0.00	<LOD	0.00	<LOD	0.00	<LOD	0.00	<LOD	0.00	<LOD	0.00	<LOD	0.00	<LOD	0.00	<LOD	0.00
9Cl-PF3ONS	<LOD	0.00	<LOD	0.00	<LOD	0.00	<LOD	0.00	<LOD	0.00	<LOD	0.00	<LOD	0.00	<LOD	0.00	<LOD	0.00	<LOD	0.00	<LOD	0.00	<LOD	0.00
11Cl-PF3OUDS	<LOD	0.00	<LOD	0.00	<LOD	0.00	<LOD	0.00	<LOD	0.00	<LOD	0.00	<LOD	0.00	<LOD	0.00	<LOD	0.00	<LOD	0.00	<LOD	0.00	<LOD	0.00
PF4OPeA	<LOD	0.00	<LOD	0.00	<LOD	0.00	<LOD	0.00	<LOD	0.00	<LOD	0.00	<LOD	0.00	<LOD	0.00	<LOD	0.00	<LOD	0.00	<LOD	0.00	<LOD	0.00
PF5OHxA	<LOD	0.00	<LOD	0.00	<LOD	0.00	<LOD	0.00	<LOD	0.00	<LOD	0.00	<LOD	0.00	<LOD	0.00	<LOD	0.00	<LOD	0.00	<LOD	0.00	<LOD	0.00
3-6-OPFHpA	<LOD	0.00	<LOD	0.00	<LOD	0.00	<LOD	0.00	<LOD	0.00	<LOD	0.00	<LOD	0.00	<LOD	0.00	<LOD	0.00	<LOD	0.00	<LOD	0.00	<LOD	0.00
PFEESA	<LOD	0.00	<LOD	0.00	<LOD	0.00	<LOD	0.00	<LOD	0.00	<LOD	0.00	<LOD	0.00	<LOD	0.00	<LOD	0.00	<LOD	0.00	<LOD	0.00	<LOD	0.00
10:2FTS	<LOD	0.00	<LOD	0.00	<LOD	0.00	<LOD	0.00	<LOD	0.00	<LOD	0.00	<LOD	0.00	<LOD	0.00	<LOD	0.00	<LOD	0.00	<LOD	0.00	<LOD	0.00
PFECHS	0.48	0.01	0.64	0.06	0.13	0.00	0.47	0.04	1.11	0.00	1.13	0.03	1.40	0.05	1.74	0.14	0.18	0.01	0.42	0.02	0.16	0.02	0.46	0.03
4:2FTA	<LOD	0.00	<LOD	0.00	<LOD	0.00	<LOD	0.00	<LOD	0.00	<LOD	0.00	<LOD	0.00	<LOD	0.00	<LOD	0.00	<LOD	0.00	<LOD	0.00	<LOD	0.00
6:2FTA	<LOD	0.00	<LOD	0.00	<LOD	0.00	<LOD	0.00	<LOD	0.00	<LOD	0.00	<LOD	0.00	<LOD	0.00	<LOD	0.00	<LOD	0.00	<LOD	0.00	<LOD	0.00

8:2FTA	<LOD	0.00	<LOD	0.00	<LOD	0.00	<LOD	0.00	<LOD	0.00	<LOD	0.00	<LOD	0.00	<LOD	0.00	<LOD	0.00	<LOD	0.00	<LOD	0.00	<LOD	0.00
6:2diPAP	<LOD	0.00	<LOD	0.00	<LOD	0.00	<LOD	0.00	<LOD	0.00	<LOD	0.00	<LOD	0.00	<LOD	0.00	<LOD	0.00	<LOD	0.00	<LOD	0.00	<LOD	0.00
6:6PFPi	<LOD	0.00	<LOD	0.00	<LOD	0.00	<LOD	0.00	<LOD	0.00	<LOD	0.00	<LOD	0.00	<LOD	0.00	<LOD	0.00	<LOD	0.00	<LOD	0.00	<LOD	0.00
8:2PAP	<LOD	0.00	<LOD	0.00	<LOD	0.00	<LOD	0.00	<LOD	0.00	<LOD	0.00	<LOD	0.00	<LOD	0.00	<LOD	0.00	<LOD	0.00	<LOD	0.00	<LOD	0.00
EtFOSA	<LOD	0.00	<LOD	0.00	<LOD	0.00	<LOD	0.00	<LOD	0.00	<LOD	0.00	<LOD	0.00	<LOD	0.00	<LOD	0.00	<LOD	0.00	<LOD	0.00	<LOD	0.00
MeFOSA	<LOD	0.00	<LOD	0.00	<LOD	0.00	<LOD	0.00	<LOD	0.00	<LOD	0.00	<LOD	0.00	<LOD	0.00	<LOD	0.00	<LOD	0.00	<LOD	0.00	<LOD	0.00
MeFOSE	<LOD	0.00	<LOD	0.00	<LOD	0.00	<LOD	0.00	<LOD	0.00	<LOD	0.00	<LOD	0.00	<LOD	0.00	<LOD	0.00	<LOD	0.00	<LOD	0.00	<LOD	0.00
EtFOSE	<LOD	0.00	<LOD	0.00	<LOD	0.00	<LOD	0.00	<LOD	0.00	<LOD	0.00	<LOD	0.00	<LOD	0.00	<LOD	0.00	<LOD	0.00	<LOD	0.00	<LOD	0.00
PFHO-DA	0.55	0.00	0.35	0.00	0.11	0.00	<LOD	0.00	0.78	0.00	0.42	0.00	0.65	0.00	0.35	0.00	0.46	0.00	0.44	0.00	0.40	0.00	19.03	0.00
∑Prec	1.39	0.00	1.26	0.00	0.63	0.00	0.78	0.00	2.24	0.00	1.70	0.00	2.35	0.00	2.27	0.00	0.87	0.00	2.05	0.00	0.69	0.00	19.78	0.00
Total PFAS	405.74	0.00	156.70	0.00	455.76	0.00	300.11	0.00	470.93	0.00	197.65	0.00	496.21	0.00	191.10	0.00	1146.63	0.00	168.10	0.00	519.15	0.00	270.00	0.00

	Surface water																				*Groundwater*			
	7				8				9				10				11				12			
	DW-7		RW-7		DW-8		RW-8		DW-9		RW-9		DW-10		RW-10		DW-11		RW-11		DW-12		RW-12	
	AV	ST	AV	ST	AV	ST	AV	ST	AV	ST	AV	ST	AV	ST	AV	ST	AV	ST	AV	ST	AV	ST	AV	ST
TFA	645.90	104.13	641.34	0.35	94.03	22.71	126.01	0.42	33.56	4.05	189.69	1.53	44.95	22.74	135.15	0.00	54.38	20.12	82.44	1.94	134.04	20.02	87.77	0.11
PFPrA	<LOD	0.00	<LOD	0.00	11.14	0.67	2.18	0.70	<LOD	0.00	<LOD	0.00	5.80	4.21	<LOD	0.00	1.14	0.00	<LOD	0.00	9.78	2.21	1.41	0.08
PFPrS	<LOD	0.00	<LOD	0.00	0.14	0.01	0.07	0.01	0.08	0.01	<LOD	0.00	0.09	0.01	<LOD	0.00	<LOD	0.00	<LOD	0.00	<LOD	0.00	0.07	0.00
PFEtS	<LOD	0.00	<LOD	0.00	<LOD	0.00	<LOD	0.00	<LOD	0.00	<LOD	0.00	<LOD	0.00	<LOD	0.00	<LOD	0.00	<LOD	0.00	<LOD	0.00	<LOD	0.00
∑ultrshort	645.90	0.00	641.34	0.00	105.31	0.00	128.25	0.00	33.64	0.00	189.69	0.00	50.84	0.00	135.15	0.00	55.52	0.00	82.44	0.00	143.82	0.00	89.25	0.00
PFBA	1.27	0.01	2.54	0.45	12.28	1.12	14.06	1.53	3.49	0.71	4.81	0.56	4.46	0.57	3.25	0.00	7.90	0.38	14.95	0.00	0.43	0.26	2.62	0.29
PFPeA	0.29	0.08	<LOD	0.00	3.75	0.11	2.68	0.08	0.52	0.01	0.95	0.11	2.20	0.04	1.90	0.00	1.18	0.05	2.82	0.64	<LOD	0.00	0.47	0.04
PFHxA	0.30	0.02	1.64	0.23	3.03	0.05	2.42	0.06	0.75	0.01	1.20	0.08	1.23	0.02	1.63	0.00	0.68	0.03	2.69	0.09	<LOD	0.00	0.39	0.02
PFHpA	0.24	0.00	1.21	0.12	1.98	0.03	1.70	0.05	0.68	0.06	0.92	0.07	0.37	0.01	0.83	0.00	0.32	0.04	1.83	0.02	<LOD	0.00	0.05	0.01
L-PFOA	0.74	0.00	4.10	1.08	4.02	0.06	5.81	0.11	4.28	0.50	8.46	0.40	1.60	0.03	5.62	0.00	1.07	0.01	41.97	1.17	0.06	0.01	0.10	0.01
Br-PFOA	0.09	0.00	0.30	0.01	1.28	0.30	0.81	0.01	0.77	0.07	0.88	0.01	0.35	0.01	0.42	0.00	0.34	0.01	3.85	0.12	<LOD	0.01	<LOD	0.00
∑PFOA	0.83	0.00	4.39	0.00	5.30	0.00	6.61	0.00	5.05	0.00	9.34	0.00	1.95	0.00	6.04	0.00	1.41	0.00	45.83	0.00	0.07	0.00	0.13	0.00

PFNA	0.13	0.00	0.30	0.07	0.28	0.01	0.26	0.01	0.17	0.00	0.21	0.01	<LOD	0.00	0.09	0.00	<LOD	0.00	0.42	0.01	<LOD	0.00	<LOD	0.00
PFDA	0.18	0.01	0.81	0.48	0.14	0.00	0.16	0.02	<LOD	0.00	0.05	0.00	<LOD	0.00	0.05	0.00	<LOD	0.00	0.37	0.09	<LOD	0.00	<LOD	0.00
PFUdA	<LOD	0.00	<LOD	0.00	<LOD	0.00	<LOD	0.00	<LOD	0.00	<LOD	0.00	<LOD	0.00	<LOD	0.00	<LOD	0.00	<LOD	0.00	<LOD	0.00	<LOD	0.00
PFDoA	<LOD	0.00	0.12	0.03	<LOD	0.00	<LOD	0.00	<LOD	0.00	<LOD	0.00	<LOD	0.00	<LOD	0.00	<LOD	0.00	0.16	0.02	<LOD	0.00	<LOD	0.00
PFTrDA	<LOD	0.00	<LOD	0.00	<LOD	0.00	<LOD	0.00	<LOD	0.00	<LOD	0.00	<LOD	0.00	<LOD	0.00	<LOD	0.00	<LOD	0.00	<LOD	0.00	<LOD	0.00
PFTeDA	<LOD	0.00	1.65	0.21	<LOD	0.00	3.15	1.71	<LOD	0.00	1.68	0.24	<LOD	0.00	<LOD	0.00	<LOD	0.00	<LOD	0.00	<LOD	0.00	<LOD	0.00
∑PFCA	3.25	0.00	17.06	0.00	26.76	0.00	37.66	0.00	10.70	0.00	28.50	0.00	10.24	0.00	19.85	0.00	11.49	0.00	114.89	0.00	0.50	0.00	3.79	0.00
PFBS	0.22	0.01	0.09	0.04	3.50	0.23	5.13	0.44	1.74	0.03	2.44	0.06	1.69	0.01	5.06	0.00	0.81	0.02	19.00	0.19	0.06	0.01	0.10	0.01
PFPeS	0.09	0.00	0.18	0.02	0.38	0.03	0.35	0.01	0.19	0.01	0.18	0.00	0.09	0.00	0.18	0.00	<LOD	0.00	0.62	0.07	0.09	0.01	0.17	0.01
L-PFHxS	0.15	0.00	0.52	0.00	1.76	0.03	2.03	0.04	0.38	0.03	0.43	0.00	<LOD	0.00	0.41	0.00	0.04	0.00	0.63	0.01	0.04	0.00	0.06	0.00
Br-PFHxS	0.03	0.00	0.09	0.00	0.41	0.05	0.33	0.01	0.11	0.01	0.12	0.00	<LOD	0.00	0.14	0.00	<LOD	0.00	0.26	0.01	<LOD	0.00	0.03	0.00
∑PFHxS	0.18	0.00	0.61	0.00	2.17	0.00	2.36	0.00	0.49	0.00	0.55	0.00	0.04	0.00	0.55	0.00	0.05	0.00	0.88	0.00	0.05	0.00	0.09	0.00
PFHpS	<LOD	0.00	<LOD	0.00	0.13	0.05	0.08	0.00	0.05	0.05	<LOD	0.00	<LOD	0.00	<LOD	0.00	<LOD	0.00	<LOD	0.00	<LOD	0.00	<LOD	0.00
Br-PFHpS	<LOD	0.00	<LOD	0.00	<LOD	0.00	<LOD	0.01	<LOD	0.00	<LOD	0.00	<LOD	0.00	<LOD	0.00	<LOD	0.00	<LOD	0.00	<LOD	0.00	<LOD	0.00
∑PFHpS	<LOD	0.00	<LOD	0.00	0.13	0.00	0.08	0.00	0.05	0.00	<LOD	0.00	<LOD	0.00	<LOD	0.00	<LOD	0.00	<LOD	0.00	<LOD	0.00	<LOD	0.00
L-PFOS	0.13	0.00	0.46	0.00	1.62	0.07	2.25	0.00	0.37	0.20	0.54	0.00	0.05	0.01	0.71	0.00	0.04	0.02	0.79	0.02	<LOD	0.01	<LOD	0.00
Br-PFOS	0.20	0.00	0.42	0.09	1.86	0.36	1.34	0.25	0.50	0.04	0.56	0.03	0.10	0.02	0.76	0.00	0.03	0.01	1.05	0.02	<LOD	0.00	<LOD	0.00
∑PFOS	0.33	0.00	0.88	0.00	3.48	0.00	3.59	0.00	0.87	0.00	1.10	0.00	0.15	0.00	1.47	0.00	0.06	0.00	1.84	0.00	<LOD	0.00	<LOD	0.00
PFNS	<LOD	0.00	<LOD	0.00	<LOD	0.00	<LOD	0.00	<LOD	0.00	<LOD	0.00	<LOD	0.00	<LOD	0.00	<LOD	0.00	<LOD	0.00	<LOD	0.00	<LOD	0.00
PFDS	<LOD	0.00	<LOD	0.00	<LOD	0.00	<LOD	0.00	<LOD	0.00	<LOD	0.00	<LOD	0.00	<LOD	0.00	<LOD	0.00	<LOD	0.00	<LOD	0.00	<LOD	0.00
∑PFSA	0.81	0.00	3.25	0.00	9.66	0.00	17.54	0.00	3.34	0.00	5.92	0.00	1.97	0.00	9.27	0.00	0.92	0.00	25.07	0.00	0.23	0.00	0.46	0.00
FBSA	<LOD	0.00	<LOD	0.00	<LOD	0.00	<LOD	0.00	<LOD	0.02	<LOD	0.00	<LOD	0.01	<LOD	0.00	<LOD	0.00	<LOD	0.00	<LOD	0.00	<LOD	0.00
FHxSA	<LOD	0.00	<LOD	0.00	<LOD	0.00	<LOD	0.00	<LOD	0.00	<LOD	0.00	<LOD	0.00	<LOD	0.00	<LOD	0.00	<LOD	0.00	<LOD	0.00	<LOD	0.00
FOSA	<LOD	0.00	<LOD	0.00	<LOD	0.00	<LOD	0.00	<LOD	0.00	<LOD	0.00	<LOD	0.00	<LOD	0.00	<LOD	0.00	<LOD	0.00	<LOD	0.00	<LOD	0.00
L-MeFOSAA	<LOD	0.00	<LOD	0.00	<LOD	0.00	<LOD	0.00	<LOD	0.00	<LOD	0.00	<LOD	0.00	<LOD	0.00	<LOD	0.00	<LOD	0.00	<LOD	0.00	<LOD	0.00
Br-MeFOSAA	<LOD	0.00	<LOD	0.00	<LOD	0.00	<LOD	0.00	<LOD	0.00	<LOD	0.00	<LOD	0.00	<LOD	0.00	<LOD	0.00	0.11	0.01	<LOD	0.00	<LOD	0.00
L-EtFOSAA	<LOD	0.00	<LOD	0.00	<LOD	0.00	<LOD	0.00	<LOD	0.00	<LOD	0.00	<LOD	0.00	0.07	0.00	<LOD	0.00	0.32	0.03	<LOD	0.00	<LOD	0.00
Br-EtFOSAA	<LOD	0.00	<LOD	0.00	<LOD	0.00	<LOD	0.00	<LOD	0.00	<LOD	0.00	<LOD	0.00	0.13	0.00	<LOD	0.00	0.47	0.03	<LOD	0.00	<LOD	0.00
4:2FTS	<LOD	0.00	<LOD	0.00	<LOD	0.00	<LOD	0.00	<LOD	0.00	<LOD	0.00	<LOD	0.00	<LOD	0.00	<LOD	0.00	<LOD	0.00	<LOD	0.00	<LOD	0.00

6:2FTS	<LOD	0.00	<LOD	0.00	<LOD	0.01	0.28	0.02	<LOD	0.00	0.17	0.02	<LOD	0.01	0.26	0.00	<LOD	0.00	0.68	0.05	<LOD	0.00	<LOD	0.00
8:2FTS	0.17	0.00	1.58	0.39	<LOD	0.00	<LOD	0.00	<LOD	0.00	<LOD	0.00	<LOD	0.00	0.08	0.00	<LOD	0.00	0.42	0.04	<LOD	0.00	<LOD	0.00
ADONA	<LOD	0.00	<LOD	0.00	<LOD	0.00	<LOD	0.00	<LOD	0.00	<LOD	0.00	<LOD	0.00	<LOD	0.00	<LOD	0.00	0.20	0.00	<LOD	0.00	<LOD	0.00
9Cl-PF3ONS	<LOD	0.00	<LOD	0.00	<LOD	0.00	<LOD	0.00	<LOD	0.00	<LOD	0.00	<LOD	0.00	<LOD	0.00	<LOD	0.00	<LOD	0.00	<LOD	0.00	<LOD	0.00
11Cl-PF3OUDS	<LOD	0.00	<LOD	0.00	<LOD	0.00	<LOD	0.00	<LOD	0.00	<LOD	0.00	<LOD	0.00	<LOD	0.00	<LOD	0.00	<LOD	0.00	<LOD	0.00	<LOD	0.00
PF4OPeA	<LOD	0.00	<LOD	0.00	<LOD	0.00	<LOD	0.00	<LOD	0.00	<LOD	0.00	<LOD	0.00	<LOD	0.00	<LOD	0.00	<LOD	0.00	<LOD	0.00	<LOD	0.00
PF5OHxA	<LOD	0.00	<LOD	0.00	<LOD	0.00	<LOD	0.00	<LOD	0.00	<LOD	0.00	<LOD	0.00	<LOD	0.00	<LOD	0.00	<LOD	0.00	<LOD	0.00	<LOD	0.00
3-6-OPFHpA	<LOD	0.00	<LOD	0.00	<LOD	0.00	<LOD	0.00	<LOD	0.00	<LOD	0.00	<LOD	0.00	<LOD	0.00	<LOD	0.02	<LOD	0.00	<LOD	0.00	<LOD	0.00
PFEESA	<LOD	0.00	<LOD	0.00	<LOD	0.00	<LOD	0.00	<LOD	0.00	<LOD	0.00	<LOD	0.00	<LOD	0.00	<LOD	0.00	<LOD	0.00	<LOD	0.00	<LOD	0.00
10:2FTS	0.31	0.06	4.93	1.04	<LOD	0.00	<LOD	0.00	<LOD	0.00	<LOD	0.00	<LOD	0.00	0.07	0.00	<LOD	0.00	<LOD	0.00	<LOD	0.00	<LOD	0.00
PFECHS	0.06	0.00	0.15	0.00	0.52	0.09	0.56	0.05	0.29	0.03	0.56	0.01	<LOD	0.00	0.14	0.00	0.06	0.00	0.23	0.01	<LOD	0.00	<LOD	0.00
4:2FTA	<LOD	0.00	<LOD	0.00	<LOD	0.00	<LOD	0.00	<LOD	0.00	<LOD	0.00	<LOD	0.00	<LOD	0.00	<LOD	0.00	<LOD	0.00	<LOD	0.00	<LOD	0.00
6:2FTA	<LOD	0.00	<LOD	0.00	<LOD	0.00	<LOD	0.00	<LOD	0.00	<LOD	0.00	<LOD	0.00	<LOD	0.00	<LOD	0.00	<LOD	0.00	<LOD	0.00	<LOD	0.00
8:2FTA	<LOD	0.00	<LOD	0.00	<LOD	0.00	<LOD	0.00	<LOD	0.00	<LOD	0.00	<LOD	0.00	<LOD	0.00	<LOD	0.00	<LOD	0.00	<LOD	0.00	<LOD	0.00
6:2diPAP	<LOD	0.00	<LOD	0.00	<LOD	0.00	<LOD	0.00	<LOD	0.00	<LOD	0.00	<LOD	0.00	<LOD	0.00	<LOD	0.00	<LOD	0.00	<LOD	0.00	<LOD	0.00
6:6PFPi	<LOD	0.00	2.51	0.22	<LOD	0.00	0.79	0.02	<LOD	0.00	1.44	0.05	<LOD	0.00	0.62	0.00	<LOD	0.00	<LOD	0.00	<LOD	0.00	<LOD	0.00
8:2PAP	<LOD	0.00	<LOD	0.00	<LOD	0.00	<LOD	0.00	<LOD	0.00	<LOD	0.00	<LOD	0.00	<LOD	0.00	<LOD	0.00	<LOD	0.00	<LOD	0.00	<LOD	0.00
EtFOSA	<LOD	0.00	0.97	0.10	<LOD	0.00	<LOD	0.00	<LOD	0.00	<LOD	0.00	<LOD	0.00	<LOD	0.00	<LOD	0.00	<LOD	0.00	<LOD	0.00	<LOD	0.00
MeFOSA	<LOD	0.00	<LOD	0.00	<LOD	0.00	<LOD	0.00	<LOD	0.00	<LOD	0.00	<LOD	0.00	<LOD	0.00	<LOD	0.00	<LOD	0.00	<LOD	0.00	<LOD	0.00
MeFOSE	<LOD	0.00	<LOD	0.00	<LOD	0.00	<LOD	0.00	<LOD	0.00	<LOD	0.00	<LOD	0.00	<LOD	0.00	<LOD	0.00	<LOD	0.00	<LOD	0.00	<LOD	0.00
EtFOSE	<LOD	0.00	<LOD	0.00	<LOD	0.00	<LOD	0.00	<LOD	0.00	<LOD	0.00	<LOD	0.00	<LOD	0.00	<LOD	0.00	<LOD	0.00	<LOD	0.00	<LOD	0.00
PFHO-DA	<LOD	0.00	<LOD	0.00	<LOD	0.00	<LOD	0.00	<LOD	0.00	0.19	0.00	9.83	0.00	9.60	0.00	<LOD	0.00	<LOD	0.00	<LOD	0.00	<LOD	0.00
∑Prec	0.54	0.00	10.14	0.00	0.54	0.00	1.63	0.00	0.34	0.00	2.36	0.00	9.90	0.00	10.98	0.00	0.25	0.00	2.43	0.00	<LOD	0.00	<LOD	0.00
Total PFAS	650.51	0.00	671.79	0.00	142.27	0.00	185.09	0.00	48.02	0.00	226.47	0.00	72.95	0.00	175.25	0.00	68.18	0.00	224.83	0.00	144.56	0.00	93.50	0.00

Groundwater

	13				14				15				16				17				18			
	DW-13		RW-13		DW-14		RW-14		DW-15		RW-15		DW-16		RW-16		DW-17		RW-17		DW-18		RW-18	
	AV	ST	AV	ST	AV	ST	AV	ST	AV	ST	AV	ST	AV	ST	AV	ST	AV	ST	AV	ST	AV	ST	AV	ST
TFA	482.95	124.32	520.93	6.28	384.88	9.94	388.15	1.93	438.09	63.03	483.63	0.19	98.19	3.25	204.46	0.92	90.58	12.61	352.49	8.04	88.44	0.07	133.73	1.14
PFPrA	<LOD	0.00	<LOD	0.00	<LOD	0.00	<LOD	0.00	<LOD	0.00	<LOD	0.00	<LOD	0.00	4.90	1.96	28.39	0.69	11.13	1.18	8.99	1.27	1.67	0.33
PFPrS	<LOD	0.00	<LOD	0.00	<LOD	0.00	<LOD	0.00	<LOD	0.00	<LOD	0.00	<LOD	0.00	<LOD	0.00	<LOD	0.00	<LOD	0.00	<LOD	0.00	<LOD	0.00
PFEtS	<LOD	0.00	<LOD	0.00	<LOD	0.00	<LOD	0.00	<LOD	0.00	<LOD	0.00	<LOD	0.00	<LOD	0.00	<LOD	0.00	<LOD	0.00	<LOD	0.00	<LOD	0.00
∑ultrshort	482.95	0.00	520.93	0.00	384.88	0.00	388.15	0.00	438.09	0.00	483.63	0.00	98.19	0.00	209.36	0.00	118.97	0.00	363.62	0.00	97.43	0.00	135.40	0.00
PFBA	0.40	0.01	1.04	0.00	0.31	0.07	<LOD	0.00	0.13	0.03	<LOD	0.00	0.30	0.01	1.22	0.20	1.17	0.03	3.69	0.31	0.97	0.04	0.84	0.05
PFPeA	0.23	0.02	0.54	0.04	0.19	0.02	<LOD	0.00	0.10	0.01	0.31	0.01	<LOD	0.00	0.25	0.07	<LOD	0.00	0.33	0.04	0.30	0.01	<LOD	0.00
PFHxA	0.35	0.01	0.54	0.01	0.24	0.01	0.40	0.04	0.15	0.01	0.41	0.10	<LOD	0.00	0.40	0.07	<LOD	0.00	0.44	0.10	0.23	0.01	0.19	0.01
PFHpA	0.22	0.00	0.32	0.01	0.16	0.01	0.23	0.02	0.11	0.00	0.21	0.04	<LOD	0.00	0.26	0.07	<LOD	0.00	0.33	0.13	0.10	0.00	<LOD	0.00
L-PFOA	0.87	0.01	1.27	0.01	0.61	0.00	1.24	0.11	0.34	0.05	0.67	0.28	0.10	0.01	0.69	0.25	0.28	0.02	0.84	0.07	0.35	0.02	0.16	0.02
Br-PFOA	0.15	0.01	0.20	0.01	0.06	0.00	<LOD	0.00	0.05	0.01	0.08	0.01	<LOD	0.00	<LOD	0.00	0.06	0.01	0.14	0.00	<LOD	0.00	<LOD	0.00
∑PFOA	1.03	0.00	1.47	0.00	0.68	0.00	1.27	0.00	0.39	0.00	0.75	0.00	0.13	0.00	0.73	0.00	0.34	0.00	0.97	0.00	0.36	0.00	0.19	0.00
PFNA	0.09	0.00	0.10	0.00	0.08	0.00	0.05	0.01	<LOD	0.00	<LOD	0.00	<LOD	0.00	<LOD	0.02	<LOD	0.00	<LOD	0.00	0.06	0.00	<LOD	0.00
PFDA	0.08	0.00	<LOD	0.00	0.28	0.00	0.64	0.05	0.15	0.00	0.18	0.08	<LOD	0.00	0.40	0.17	<LOD	0.00	0.07	0.01	0.13	0.01	<LOD	0.00
PFUdA	<LOD	0.00	<LOD	0.00	<LOD	0.00	0.07	0.00	<LOD	0.00	<LOD	0.00	<LOD	0.00	<LOD	0.00	<LOD	0.00	<LOD	0.00	<LOD	0.00	<LOD	0.00
PFDoA	<LOD	0.00	<LOD	0.00	<LOD	0.01	0.09	0.00	<LOD	0.00	<LOD	0.01	<LOD	0.00	0.05	0.05	<LOD	0.00	<LOD	0.00	<LOD	0.00	<LOD	0.00
PFTrDA	<LOD	0.00	<LOD	0.00	<LOD	0.00	<LOD	0.00	<LOD	0.00	<LOD	0.00	<LOD	0.00	<LOD	0.00	<LOD	0.00	<LOD	0.00	<LOD	0.00	<LOD	0.00
PFTeDA	<LOD	0.00	<LOD	0.00	<LOD	0.00	0.55	0.05	<LOD	0.00	<LOD	0.00	<LOD	0.00	<LOD	0.00	<LOD	0.00	<LOD	0.00	<LOD	0.00	<LOD	0.00
∑PFCA	2.39	0.00	5.47	0.00	1.97	0.00	4.57	0.00	1.03	0.00	2.61	0.00	0.44	0.00	4.02	0.00	1.51	0.00	6.80	0.00	2.17	0.00	1.41	0.00
PFBS	0.12	0.00	<LOD	0.00	<LOD	0.00	<LOD	0.00	<LOD	0.00	<LOD	0.00	0.05	0.00	<LOD	0.00	0.07	0.00	<LOD	0.00	<LOD	0.00	<LOD	0.00
PFPeS	0.08	0.00	0.07	0.00	0.05	0.00	<LOD	0.00	<LOD	0.03	<LOD	0.00	<LOD	0.00	<LOD	0.00	<LOD	0.00	0.08	0.00	<LOD	0.00	<LOD	0.00
L-PFHxS	0.04	0.00	0.07	0.00	<LOD	0.00	<LOD	0.00	<LOD	0.00	<LOD	0.00	<LOD	0.00	<LOD	0.00	<LOD	0.00	<LOD	0.00	<LOD	0.00	<LOD	0.00
Br-PFHxS	<LOD	0.00	0.04	0.00	<LOD	0.00	<LOD	0.00	<LOD	0.00	<LOD	0.00	<LOD	0.00	<LOD	0.00	<LOD	0.00	<LOD	0.00	<LOD	0.00	<LOD	0.00
∑PFHxS	0.06	0.00	0.11	0.00	<LOD	0.00	<LOD	0.00	<LOD	0.00	<LOD	0.00	<LOD	0.00	<LOD	0.00	<LOD	0.00	<LOD	0.00	<LOD	0.00	<LOD	0.00
PFHpS	<LOD	0.00	<LOD	0.00	<LOD	0.00	<LOD	0.00	<LOD	0.00	<LOD	0.00	<LOD	0.00	<LOD	0.00	<LOD	0.00	<LOD	0.00	<LOD	0.00	<LOD	0.00
Br-PFHpS	<LOD	0.00	<LOD	0.00	<LOD	0.00	<LOD	0.00	<LOD	0.00	<LOD	0.00	<LOD	0.00	<LOD	0.00	<LOD	0.00	<LOD	0.00	<LOD	0.00	<LOD	0.00

∑PFHpS	<LOD	0.00	<LOD	0.00	<LOD	0.00	<LOD	0.00	<LOD	0.00	<LOD	0.00	<LOD	0.00	<LOD	0.00	<LOD	0.00	<LOD	0.00	<LOD	0.00	<LOD	0.00
L-PFOS	0.12	0.01	0.24	0.00	0.08	0.00	0.10	0.00	0.09	0.02	0.05	0.00	0.04	0.01	0.11	0.00	0.06	0.02	0.05	0.00	0.05	0.00	<LOD	0.00
Br-PFOS	0.17	0.00	0.31	0.01	0.09	0.00	0.09	0.00	0.08	0.01	0.07	0.02	0.02	0.01	0.10	0.03	0.06	0.02	0.11	0.01	0.02	0.00	0.06	0.00
∑PFOS	0.29	0.00	0.54	0.00	0.16	0.00	0.20	0.00	0.17	0.00	0.13	0.00	0.06	0.00	0.21	0.00	0.12	0.00	0.16	0.00	0.07	0.00	0.06	0.00
PFNS	<LOD	0.00	<LOD	0.00	<LOD	0.00	<LOD	0.00	<LOD	0.00	<LOD	0.00	<LOD	0.00	<LOD	0.02	<LOD	0.00	<LOD	0.01	<LOD	0.00	<LOD	0.00
PFDS	<LOD	0.00	<LOD	0.00	<LOD	0.00	<LOD	0.00	<LOD	0.00	<LOD	0.00	<LOD	0.00	<LOD	0.00	<LOD	0.00	<LOD	0.00	<LOD	0.00	<LOD	0.00
∑PFSA	0.55	0.00	1.38	0.00	0.26	0.00	0.39	0.00	0.23	0.00	0.25	0.00	0.10	0.00	0.42	0.00	0.19	0.00	0.39	0.00	0.11	0.00	0.12	0.00
FBSA	<LOD	0.00	<LOD	0.00	<LOD	0.00	<LOD	0.00	<LOD	0.00	<LOD	0.00	<LOD	0.00	<LOD	0.00	<LOD	0.00	<LOD	0.00	<LOD	0.00	<LOD	0.00
FHxSA	<LOD	0.00	<LOD	0.00	<LOD	0.00	<LOD	0.00	<LOD	0.00	<LOD	0.00	<LOD	0.00	<LOD	0.00	<LOD	0.00	<LOD	0.00	<LOD	0.00	<LOD	0.00
FOSA	<LOD	0.00	<LOD	0.00	<LOD	0.00	<LOD	0.00	<LOD	0.00	<LOD	0.00	<LOD	0.00	<LOD	0.00	<LOD	0.00	<LOD	0.00	<LOD	0.00	<LOD	0.00
L-MeFOSAA	<LOD	0.00	<LOD	0.00	<LOD	0.00	<LOD	0.00	<LOD	0.00	<LOD	0.00	<LOD	0.00	<LOD	0.00	<LOD	0.00	<LOD	0.00	<LOD	0.00	<LOD	0.00
Br-MeFOSAA	<LOD	0.00	<LOD	0.00	<LOD	0.00	<LOD	0.00	<LOD	0.00	<LOD	0.00	<LOD	0.00	<LOD	0.00	<LOD	0.00	<LOD	0.00	<LOD	0.00	<LOD	0.00
L-EtFOSAA	<LOD	0.00	<LOD	0.00	<LOD	0.00	<LOD	0.00	<LOD	0.00	<LOD	0.00	<LOD	0.00	<LOD	0.06	<LOD	0.00	<LOD	0.00	<LOD	0.00	<LOD	0.00
Br-EtFOSAA	<LOD	0.00	<LOD	0.00	<LOD	0.00	<LOD	0.00	<LOD	0.00	<LOD	0.00	<LOD	0.00	0.06	0.08	<LOD	0.00	<LOD	0.00	<LOD	0.00	<LOD	0.00
4:2FTS	<LOD	0.00	<LOD	0.00	<LOD	0.00	<LOD	0.00	<LOD	0.00	<LOD	0.00	<LOD	0.00	<LOD	0.00	<LOD	0.00	<LOD	0.00	<LOD	0.00	<LOD	0.00
6:2FTS	<LOD	0.00	0.14	0.00	<LOD	0.00	0.15	0.01	0.05	0.01	<LOD	0.00	0.09	0.09	0.19	0.00	<LOD	0.00	0.14	0.00	<LOD	0.00	0.12	0.00
8:2FTS	0.11	0.00	0.21	0.02	0.21	0.01	0.92	0.21	0.18	0.00	1.03	0.05	<LOD	0.00	0.35	0.11	<LOD	0.00	0.14	0.04	0.20	0.01	<LOD	0.00
ADONA	<LOD	0.00	<LOD	0.00	<LOD	0.00	<LOD	0.00	<LOD	0.00	<LOD	0.00	<LOD	0.00	<LOD	0.00	<LOD	0.00	<LOD	0.00	<LOD	0.00	<LOD	0.00
9Cl-PF3ONS	<LOD	0.00	<LOD	0.00	<LOD	0.00	<LOD	0.00	<LOD	0.00	<LOD	0.00	<LOD	0.00	<LOD	0.00	<LOD	0.00	<LOD	0.00	<LOD	0.00	<LOD	0.00
11Cl-PF3OUDS	<LOD	0.00	<LOD	0.00	<LOD	0.00	<LOD	0.00	<LOD	0.00	<LOD	0.00	<LOD	0.00	<LOD	0.00	<LOD	0.00	<LOD	0.00	<LOD	0.00	<LOD	0.00
PF4OPeA	<LOD	0.00	<LOD	0.00	<LOD	0.00	<LOD	0.00	<LOD	0.00	<LOD	0.00	<LOD	0.00	<LOD	0.00	<LOD	0.00	<LOD	0.00	<LOD	0.00	<LOD	0.00
PF5OHxA	<LOD	0.00	<LOD	0.00	<LOD	0.00	<LOD	0.00	<LOD	0.00	<LOD	0.00	<LOD	0.00	<LOD	0.00	<LOD	0.00	<LOD	0.00	<LOD	0.00	<LOD	0.00
3-6-OPFHpA	<LOD	0.00	<LOD	0.00	<LOD	0.00	<LOD	0.00	<LOD	0.00	<LOD	0.00	<LOD	0.00	<LOD	0.00	<LOD	0.00	<LOD	0.00	<LOD	0.00	<LOD	0.00
PFEESA	<LOD	0.00	<LOD	0.00	<LOD	0.00	<LOD	0.00	<LOD	0.00	<LOD	0.00	<LOD	0.00	<LOD	0.00	<LOD	0.00	<LOD	0.00	<LOD	0.00	<LOD	0.00
10:2FTS	0.32	0.02	0.37	0.10	0.66	0.13	1.60	0.40	0.46	0.02	1.95	0.73	<LOD	0.00	0.35	0.01	0.07	0.01	0.14	0.05	0.37	0.03	<LOD	0.00
PFECHS	0.05	0.00	<LOD	0.00	<LOD	0.00	<LOD	0.00	<LOD	0.00	<LOD	0.00	<LOD	0.00	<LOD	0.00	<LOD	0.00	<LOD	0.00	<LOD	0.00	<LOD	0.00
4:2FTA	<LOD	0.00	<LOD	0.00	<LOD	0.00	<LOD	0.00	<LOD	0.00	<LOD	0.00	<LOD	0.00	<LOD	0.00	<LOD	0.00	<LOD	0.00	<LOD	0.00	<LOD	0.00
6:2FTA	<LOD	0.00	<LOD	0.00	<LOD	0.00	<LOD	0.00	<LOD	0.00	<LOD	0.00	<LOD	0.00	<LOD	0.00	<LOD	0.00	<LOD	0.00	<LOD	0.00	<LOD	0.00

8:2FTA	<LOD	0.00	<LOD	0.00	<LOD	0.00	<LOD	0.00	<LOD	0.00	<LOD	0.00	<LOD	0.00	<LOD	0.00	<LOD	0.00	<LOD	0.00	<LOD	0.00	<LOD	0.00
6:2diPAP	<LOD	0.00	<LOD	0.00	<LOD	0.00	<LOD	0.00	<LOD	0.00	<LOD	0.00	<LOD	0.00	<LOD	0.00	<LOD	0.00	<LOD	0.00	<LOD	0.00	<LOD	0.00
6:6PFPi	<LOD	0.00	0.69	0.05	<LOD	0.00	0.82	0.10	<LOD	0.00	0.83	0.14	<LOD	0.00	0.84	0.08	<LOD	0.00	0.64	0.02	<LOD	0.00	0.60	0.05
8:2PAP	<LOD	0.00	<LOD	0.00	<LOD	0.00	<LOD	0.00	<LOD	0.00	<LOD	0.00	<LOD	0.00	<LOD	0.00	<LOD	0.00	<LOD	0.00	<LOD	0.00	<LOD	0.00
EtFOSA	<LOD	0.02	0.22	0.02	<LOD	0.00	0.39	0.18	<LOD	0.01	0.59	0.23	<LOD	0.00	<LOD	0.00	<LOD	0.00	<LOD	0.00	<LOD	0.00	<LOD	0.00
MeFOSA	<LOD	0.00	<LOD	0.00	<LOD	0.00	<LOD	0.00	<LOD	0.00	<LOD	0.00	<LOD	0.00	<LOD	0.00	<LOD	0.00	<LOD	0.00	<LOD	0.00	<LOD	0.00
MeFOSE	<LOD	0.00	<LOD	0.00	<LOD	0.00	<LOD	0.00	<LOD	0.00	<LOD	0.00	<LOD	0.00	<LOD	0.00	<LOD	0.00	<LOD	0.00	<LOD	0.00	<LOD	0.00
EtFOSE	<LOD	0.00	<LOD	0.00	<LOD	0.00	<LOD	0.00	<LOD	0.00	<LOD	0.00	<LOD	0.00	<LOD	0.00	<LOD	0.00	<LOD	0.00	<LOD	0.00	<LOD	0.00
PFHO-DA	<LOD	0.00	<LOD	0.00	<LOD	0.00	<LOD	0.00	<LOD	0.00	<LOD	0.00	<LOD	0.00	<LOD	0.00	<LOD	0.00	<LOD	0.00	<LOD	0.00	<LOD	0.00
∑Prec	0.57	0.00	1.62	0.00	0.97	0.00	3.87	0.00	0.80	0.00	4.40	0.00	0.09	0.00	1.79	0.00	0.07	0.00	1.06	0.00	0.56	0.00	0.72	0.00
Total PFAS	486.47	0.00	529.40	0.00	388.08	0.00	396.98	0.00	440.15	0.00	490.90	0.00	98.82	0.00	215.59	0.00	120.74	0.00	371.88	0.00	100.26	0.00	137.65	0.00

Reference

Bil, W., Zeilmaker, M., Fragki, S., Lijzen, J., Verbruggen, E., Bokkers, B., 2022. Response to Letter to the Editor on Bil et al. 2021 "Risk Assessment of Per- and Polyfluoroalkyl Substance Mixtures: A Relative Potency Factor Approach." Environmental Toxicology and Chemistry 41, 13–18. https://doi.org/10.1002/etc.5236

Bil, W., Zeilmaker, M., Fragki, S., Lijzen, J., Verbruggen, E., Bokkers, B., 2021. Risk Assessment of Per- and Polyfluoroalkyl Substance Mixtures: A Relative Potency Factor Approach. Environmental Toxicology and Chemistry 40, 859–870.

Rietjens, I.M.C.M., Schriks, M., Houtman, C.J., Dingemans, M.M.L., Wezel, A.P. van, 2022. Letter to the Editor on Bil et al. 2021 "Risk Assessment of Per- and Polyfluoroalkyl Substance Mixtures: A Relative Potency Factor Approach." Environmental Toxicology and Chemistry 41, 7–12.
